# Demographic and clinical characteristics of patients with zinc deficiency: analysis of a nationwide Japanese medical claims database

**DOI:** 10.1038/s41598-024-53202-0

**Published:** 2024-02-02

**Authors:** Hirohide Yokokawa, Yusuke Morita, Izumi Hamada, Yuji Ohta, Nobuyuki Fukui, Nao Makino, Emi Ohata, Toshio Naito

**Affiliations:** 1 Department of General Medicine, Faculty of Medicine, Juntendo University, Tokyo, Japan; 2 Department of Data Science, Nobelpharma Co. Ltd, Tokyo, Japan; 3 Department of Academic Services, 4DIN Ltd., #805 Shinbashiekimae Bldg.1 2-20-15 Shinbashi Minato-ku, Tokyo, 105-0004 Japan; 4 Center for Promotion of Data Science, Juntendo University Graduate School of Medicine, Tokyo, Japan

**Keywords:** Risk factors, Comorbidities

## Abstract

Zinc deficiency, affecting more than 2 billion people globally, poses a significant public health burden due to its numerous unfavorable effects, such as impaired immune function, taste and smell disorders, pneumonia, growth retardation, visual impairment, and skin disorders. Despite its critical role, extensive large-scale studies investigating the correlation between patient characteristics and zinc deficiency still need to be completed. We conducted a retrospective, cross-sectional observational study using a nationwide Japanese claims database from January 2019 to December 2021. The study population included 13,100 patients with available serum zinc concentration data, excluding individuals under 20 and those assessed for zinc concentrations after being prescribed zinc-containing medication. Significant associations with zinc deficiency were noted among older adults, males, and inpatients. Multivariate analysis, adjusting for age and sex, indicated significant associations with comorbidities, including pneumonitis due to solids and liquids with an adjusted Odds Ratio (aOR) of 2.959; decubitus ulcer and pressure area (aOR 2.403), sarcopenia (aOR 2.217), COVID-19 (aOR 1.889), and chronic kidney disease (aOR 1.835). Significant association with medications, including spironolactone (aOR 2.523), systemic antibacterials (aOR 2.419), furosemide (aOR 2.138), antianemic preparations (aOR 2.027), and thyroid hormones (aOR 1.864) were also found. These results may aid clinicians in identifying patients at risk of zinc deficiency, potentially improving care outcomes.

## Introduction

Zinc deficiency is a global burden, impacting more than 2 billion people all over the world^1,2^. Despite variations in prevalence, both developing and developed countries are affected; a global survey of 188 countries revealed an average prevalence rate of 17.3% and a standard deviation of 11.1%^3^. Dietary habits and the availability of zinc-rich foods may partially explain variations in prevalence. Studies suggest that in the US, red meat is a major source of zinc^4^, whereas in Japan, rice is often reported as the major source^5^ of particular concern is the increased prevalence of zinc deficiency observed in infants, pregnant women, and the elderly^6–8^.

Zinc is an essential micronutrient, crucial for many metabolic processes in cells. Zinc acts as a vital cofactor, supporting enzymes that are essential for metabolizing proteins, lipids, and nucleic acids^9^. Zinc also plays a pivotal role in immune functions, fostering the development and activity of neutrophils and natural killer cells^10^. Furthermore, zinc is fundamental during pregnancy, childhood, and adolescence, contributing to cell division and DNA synthesis^11^. Zinc is crucial for enzymes and proteins involved in taste and smell perception^12^ and significantly aids wound healing by contributing to collagen synthesis, inflammation modulation, and cell signaling and division^13^.

The wide-ranging health consequences of zinc deficiency make it a critical public health concern. Zinc deficiency increases the risk of impaired immune function, taste and smell disorders, pneumonia, growth retardation, visual impairment, skin disorders, impaired lymphocyte function, anorexia, and diarrhea^14–17^. Zinc deficiency has recently been reported to adversely affect outcomes in patients with liver disease, inflammatory bowel disease, chronic kidney disease, and those infected with COVID-19^18–21^. Long-term use of drugs known to chelate zinc can also lead to deficiency^22^, underscoring the importance of assessing patients' zinc status in a range of diseases.

Identifying and addressing zinc deficiency is vital for overall health. According to a recent umbrella review, proper zinc intake can potentially reduce risks of digestive cancers, depression, and type 2 diabetes mellitus, while enhancing respiratory health, bone formation, and blood lipid profiles^23^. Furthermore, zinc supplements effectively treat pneumonia, diarrhea, and ageusia/dysgeusia in children, subsequently reducing mortality^24^. For elderly individuals at high risk of COVID-19, who frequently exhibit dietary deficiencies, zinc supplementation can provide relief from severe diarrhea^25^, thereby preventing further zinc loss. Although this cost-effective intervention is vital, excessive zinc supplementation poses risks, such as copper deficiency^26^. Regular assessments of patients’ zinc levels are imperative, ensuring optimal health outcomes and timely interventions for those in need of treatment.

Although various diseases, clinical conditions, drugs, and demographic variables may increase zinc deficiency risk, only a few large-scale studies have comprehensively evaluated the variables affecting serum zinc concentrations^27,28^, with outcomes differing by population. Earlier studies using the US National Health and Nutrition Examination Survey (NHANES) show rising zinc intake from supplements over time^29^. NHANES data revealed associations between serum zinc concentration and demographic variables such as sex, age, pregnancy, and time of blood draw^27^. However, these studies didn’t consider the impact of medication and disease histories on serum zinc levels. A recent Japanese study using voluntary health checkup data reported a high prevalence of marginal serum zinc deficiency (46.0% in men and 38.4% in women), suggesting a link between nutritional status (including zinc intake) and serum zinc concentration^28^. More extensive data is needed for deeper insights into variables linked with zinc deficiency in Japan.

Therefore, this study aims to investigate the demographic characteristics, diseases, and medications associated with serum zinc levels across a diverse patient population, using a large-scale Japanese nationwide claims database, and to clarify the characteristics of patients who are likely to be zinc deficient.

## Methods

### Study design and data source

This retrospective, cross-sectional, observational study looked at records from 1 January 2019 through 31 December 2021 in a nationwide claims database (MDV Co, Tokyo, Japan), which contains anonymized administrative data from over 38 million patients across more than 460 hospitals representing approximately 26% of all acute care hospitals in Japan. The MDV database includes claims data and discharge abstract data gathered from both inpatient and outpatient visits. It has been the source of data for numerous previously reported studies^30,31^.

### Inclusion/exclusion criteria

Patients included in the analysis were those with at least one zinc concentration result in the MDV database during the study period. Patients under 20 years old were excluded, and for patients prescribed zinc-containing medication, data collected on the day following the initiation of zinc-containing medication were excluded from the analysis. Zinc-containing medication was defined as any medication with a generic name of polaprezinc or zinc acetate hydrate.

### Variables

The most recent zinc concentration value was selected for patients with multiple zinc measurements. Zinc concentration categories were defined using criteria based on the treatment guidelines for zinc deficiency published by The Japanese Society of Clinical Nutrition: deficiency (< 60 μg/dL), marginal deficiency (≥ 60 to < 80 μg/dL), and normal (≥ 80 μg/dL)^32^.

Data on sex, age at the selected zinc measurement, weight, and body mass index (BMI) within 30 days from the measurement, classification of inpatient or outpatient, and laboratory parameters were collected. For this study, comorbidities were defined as diseases diagnosed within 60 days prior to the zinc measurement, and medications referred to those prescribed within the same 60-day time frame. BMI was calculated by dividing body weight (kg) by height in meters squared (m^2^). Demographic and clinical variables of interest were selected with reference to the treatment guidelines for zinc deficiency^32^. Evaluated laboratory parameters included hemoglobin, total protein, albumin, liver enzymes (alanine aminotransferase [ALT] and aspartate aminotransferase [AST]), serum alkaline phosphatase, kidney function markers (creatinine, estimated glomerular filtration rate [eGFR]), lipid profile (triglycerides, total cholesterol, high-density lipoprotein [HDL], low-density lipoprotein [LDL]), serum iron, C-reactive protein (CRP), glucose, serum magnesium, and uric acid. The comorbidities of interest and their definitions based on the International Classification of Diseases Tenth Revision (ICD-10) code were presented in Supplementary Table S1, and the medications of interest and their definitions based on the Anatomical Therapeutic Chemical (ATC) code were presented in Supplementary Table S2.

### Ethical considerations

This study adhered to Chapter 1, Section 3, Part 1, Subsection (C), Item 3 of the ethical guidelines for Medical and Health Research Involving Human Subjects of the Ministry of Health, Labour and Welfare of Japan^33^. In accordance with this guideline, because this study used previously anonymized and de-identified data, an ethical review was waived, and patient informed consent was not required. Additionally, it conformed to the ethical principles outlined in the Declaration of Helsinki (Fortaleza Revision, 2013)^34^.

### Statistical methods

Data for demographic and clinical variables were summarized as mean ± standard deviation or number and proportion (percent), as the aim of this study was descriptive. Chi-square tests were used to compare the proportions of zinc deficiency between male and female participants within each age group. In addition, subgroup analyses were conducted for comorbidities and medications, stratified by age group, sex, and inpatient/outpatient status. The objective was to identify variables associated with zinc deficiency that are specific to sex, age, and inpatient/outpatient status. The correlation between serum zinc concentration and laboratory parameters was assessed using Spearman’s correlation coefficient. Additionally, the relationship between hemoglobin and serum zinc levels was examined by categorizing hemoglobin as ‘low’ or ‘normal’ according to WHO criteria^35^, and serum zinc levels as ‘deficiency’ or ‘normal or marginal deficiency.’ A 2 × 2 cross-tabulation was created to summarize the distribution of serum zinc levels within these hemoglobin categories for the entire cohort and a separate analysis was performed for each gender. Chi-square tests were then conducted to determine the significance of the observed differences in the prevalence of zinc deficiency between the ‘low’ and ‘normal’ hemoglobin groups for both the overall population and within each gender subset. Binary logistic regression was performed after categorizing serum zinc concentration into two groups (i.e., deficiency vs. normal or marginal deficiency). Univariate logistic regression analyses were performed for each demographic variable, including age, sex, and inpatient/outpatient status, to examine their individual associations with zinc deficiency. This was followed by separate univariate logistic regression analyses for each specific comorbidity and medication of interest, aimed at evaluating their individual associations with zinc deficiency. For each comorbidity and medication assessed in this analysis, individuals without the comorbidity or the medication were used as the control group for the calculation of odds ratios. Additionally, multivariate logistic regression analyses for each comorbidity and medication were conducted separately, incorporating not only the individual comorbidity or medication but also age group, sex, and interaction term between age group and sex as covariates, to identify variables independently associated with zinc deficiency. In the multivariate logistic regressions, the age group was categorized as 20 s, 30 s, 40 s, 50 s, 60 s, 70 s, and 80 years and older. Additionally, sensitivity analyses for the multivariate logistic regressions included inpatient/outpatient status as an additional covariate. Separate univariate logistic regressions were also conducted for inpatients and outpatients. Moreover, the demographic and clinical characteristics of both inpatients and outpatients were summarized respectively. All statistical analyses were carried out using SAS Ver.9.4 (SAS Institute, Cary, NC, USA).

## Results

**Figure 1:**
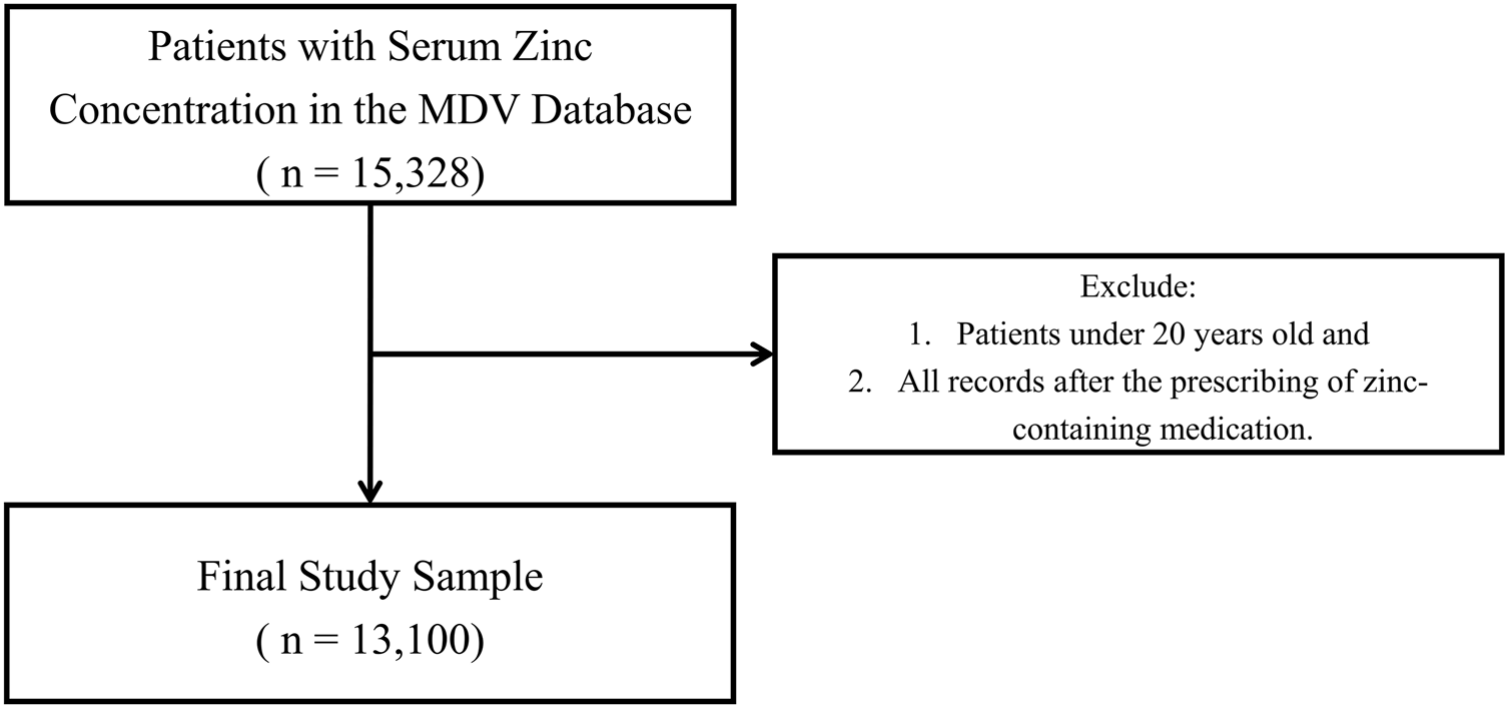
Flow Chart of Study Population.

Figure 1 presents the flow chart of the study population. Within the MDV database, serum zinc concentrations were evaluated for 15,328 patients. Among these, 13,100 were included in the final analysis.

Table 1 displays the demographic and clinical characteristics of the patients. The sex distribution was nearly equal, with 48.6% male and 51.4% female. The average age was 69.0 years. There were a considerable number of missing measurements for weight and BMI (missing percentage for weight: 63.6%, and BMI: 64.3%). The mean serum zinc concentration was 65.9 μg/dL. For serum zinc levels, 34.8% were categorized as zinc deficiency, 45.5% as marginal deficiency, and 19.7% as normal.

**Table 1:** Summary of Demographic and Clinical Characteristics.

Demographic/clinical characteristic	n (%)	Demographic/clinical characteristic	n (%)
Total patients	13,100	Hypogonadism	88 (0.7)
		Short stature	81 (0.6)
Serum zinc (μg/dL)		Hyperlipidemia	1252 (9.6)
Mean ± SD (n)	65.9 ± 17.6 (n = 13,100)	Hypertensive diseases	2447 (18.7)
Serum zinc level		Acute myocardial infarction	873 (6.7)
Deficiency (< 60 μg/dL)	4557 (34.8)	Atrial fibrillation and flutter	662 (5.1)
Marginal (≥ 60 to < 80 μg/dL)	5964 (45.5)	Heart failure	2365 (18.1)
Normal (≥ 80 μg/dL)	2579 (19.7)	Cerebrovascular diseases	1331 (10.2)
		Influenza and pneumonia	2088 (15.9)
Sex		COVID-19	3640 (27.8)
Male	6372 (48.6)	Pneumonitis due to solids and liquids	439 (3.4)
Female	6728 (51.4)	Stomatitis and related lesions	247 (1.9)
		Liver disease	2657 (20.3)
Age (years) (on serum zinc-measurement day)		Dermatitis and eczema	645 (4.9)
Mean ± SD (n)	69.0 ± 17.1 (n = 13,100)	Alopecia areata	76 (0.6)
Age group (on serum zinc-measurement day)		Decubitus ulcer and pressure area	265 (2.0)
20–29 years	492 (3.8)	Muscle wasting and atrophy, not elsewhere classified (Sarcopenia)	1166 (8.9)
30–39 years	529 (4.0)	Osteoporosis	823 (6.3)
40–49 years	901 (6.9)	Chronic kidney disease	1321 (10.1)
50–59 years	1393 (10.6)	Disturbances of smell and taste	492 (3.8)
60–69 years	2049 (15.6)	Anorexia	296 (2.3)
70–79 years	3658 (27.9)	Injuries to the head	185 (1.4)
≥ 80 years	4078 (31.1)	Fracture	834 (6.4)
Weight (kg)			
Mean ± SD (n)	54.4 ± 14.2 (n = 4772)	Medication (within 60 days before serum zinc-measurement)	
BMI (kg/m^2^)		Antihyperglycemics	2191 (16.7)
Mean ± SD (n)	21.7 ± 4.5 (n = 4676)	Antihypertensive agents	5053 (38.6)
BMI group		Spironolactone	818 (6.2)
< 25	3750 (28.6)	Furosemide	2081 (15.9)
≥ 25	926 (7.1)	ACE inhibitors	324 (2.5)
Missing	8424 (64.3)	Angiotensin II receptor blockers	1862 (14.2)
		Antihyperlipidemics	2148 (16.4)
Inpatient/Outpatient		Statins	1861 (14.2)
Inpatient	5614 (42.9)	Antithrombotic agents	3859 (29.5)
Outpatient	7420 (56.6)	H2 blockers	930 (7.1)
Missing	66 (0.5)	Proton pump inhibitors	4273 (32.6)
		Antianemic preparations	1935 (14.8)
Comorbidity (within 60 days before serum zinc-measurement)		Corticosteroids	1125 (8.6)
Intestinal infectious diseases	639 (4.9)	Thyroid hormones	410 (3.1)
Noninfective enteritis and colitis	135 (1.0)	Systemic antibacterials	3981 (30.4)
Tuberculosis	283 (2.2)	Drugs for treatment of bone diseases	484 (3.7)
Malignant neoplasms of digestive organs	2536 (19.4)	Anti-Parkinson agents	215 (1.6)
Nutritional anemias	4144 (31.6)	Antipsychotics	1097 (8.4)
Diabetes mellitus	4224 (32.2)	Anxiolytics	2442 (18.6)

*ACE* angiotensin-converting enzyme.

Table 2 displays serum zinc levels categorized by demographic characteristics. The proportion of individuals with zinc deficiency was higher in males at 36.6%, compared to females at 33.1%. Additionally, the prevalence of zinc deficiency was observed to increase with age: 31.2% in individuals in their 60 s, 35.5% in those in their 70 s, and 45.8% in those aged 80 and above. Furthermore, inpatients exhibited a higher prevalence of zinc deficiency, standing at 50.3%, compared to outpatients at 23.1%. Table 2 also presents the odds ratios (ORs) of demographic variables associated with zinc deficiency. Male sex was associated with higher odds of zinc deficiency (OR 1.165 vs female), as is older age (OR 1.301 per 10 years increase), BMI under 25 (OR 1.443 vs BMI greater than or equal to 25), and inpatient status (OR 3.367 vs outpatient), with all associations being statistically significant (*p* < 0.001).

**Table 2:** Summary of Serum Zinc Level by Demographic Characteristics.

	Serum zinc level				Zinc deficiency (< 60 μg/dL)		
	Overall	Deficiency	Marginal	Normal	Odds Ratio	95% CI	*p*-value
	n	n (%)	n (%)	n (%)			
Total patients							
–	13,100	4557 (34.8)	5964 (45.5)	2579 (19.7)			
Serum zinc							
Mean ± SD (n)	65.9 ± 17.6 (n = 13,100)	48.0 ± 9.5 (n = 4557)	69.1 ± 5.5 (n = 5964)	90.4 ± 12.4 (n = 2579)			
Sex							
Male	6372	2330 (36.6)	2789 (43.8)	1253 (19.7)	1.165	(1.084, 1.252)	< .001
Female	6728	2227 (33.1)	3175 (47.2)	1326 (19.7)	Ref		
Age (years) (on serum-zinc measurement day)							
Mean ± SD (n)	69.0 ± 17.1 (n = 13,100)	73.5 ± 15.3 (n = 4557)	67.9 ± 17.3 (n = 5964)	63.4 ± 17.7 (n = 2579)	1.301 (Age/10)	(1.270, 1.332)	< .001
Age group (on serum-zinc measurement day)							
20–29 years	492	80 (16.3)	252 (51.2)	160 (32.5)	Ref		
30–39 years	529	106 (20.0)	280 (52.9)	143 (27.0)	1.290	(0.937, 1.778)	0.119
40–49 years	901	205 (22.8)	420 (46.6)	276 (30.6)	1.517	(1.140, 2.018)	0.004
50–59 years	1393	358 (25.7)	646 (46.4)	389 (27.9)	1.781	(1.363, 2.329)	< .001
60–69 years	2049	640 (31.2)	943 (46.0)	466 (22.7)	2.339	(1.809, 3.025)	< .001
70–79 years	3658	1300 (35.5)	1697 (46.4)	661 (18.1)	2.839	(2.214, 3.641)	< .001
≥ 80 years	4078	1868 (45.8)	1726 (42.3)	484 (11.9)	4.353	(3.399, 5.574)	< .001
Weight(kg)							
Mean ± SD (n)	54.4 ± 14.2 (n = 4772)	52.5 ± 13.7 (n = 2404)	55.9 ± 14.1 (n = 1702)	57.6 ± 15.4 (n = 666)	0.906 (Weight/5)	(0.887, 0.925)	< .001
BMI(kg/m^2^)							
Mean ± SD (n)	21.7 ± 4.5 (n = 4676)	21.2 ± 4.4 (n = 2347)	22.1 ± 4.4 (n = 1672)	22.5 ± 4.8 (n = 657)	0.903 (BMI/2)	(0.879, 0.927)	< .001
BMI group							
< 25	3750	1950 (52.0)	1307 (34.9)	493 (13.1)	1.443	(1.249, 1.669)	< .001
﻿≥ 25	926	397 (42.9)	365 (39.4)	164 (17.7)	Ref		
Missing	8424	2210 (26.2)	4292 (50.9)	1922 (22.8)	0.474	(0.413, 0.545)	< .001
Smoking							
No	2897	1481 (51.1)	1033 (35.7)	383 (13.2)	Ref		
Yes	1723	844 (49.0)	628 (36.4)	251 (14.6)	0.918	(0.815, 1.034)	0.160
Missing	8480	2232 (26.3)	4303 (50.7)	1945 (22.9)	0.342	(0.313, 0.373)	< .001
Inpatients/outpatients							
Inpatient	5614	2822 (50.3)	1995 (35.5)	797 (14.2)	3.367	(3.123, 3.630)	< .001
Outpatient	7420	1713 (23.1)	3938 (53.1)	1769 (23.8)	Ref		
Missing	66	22 (33.3)	31 (47.0)	13 (19.7)	1.666	(0.996, 2.787)	0.052

*CI* confidence interval.

Serum Zinc Level: Deficiency, < 60 μg/dL; Marginal, ≥ 60 to < 80 μg/dL; Normal, ≥ 80 μg/dL.

**Figure 2:**
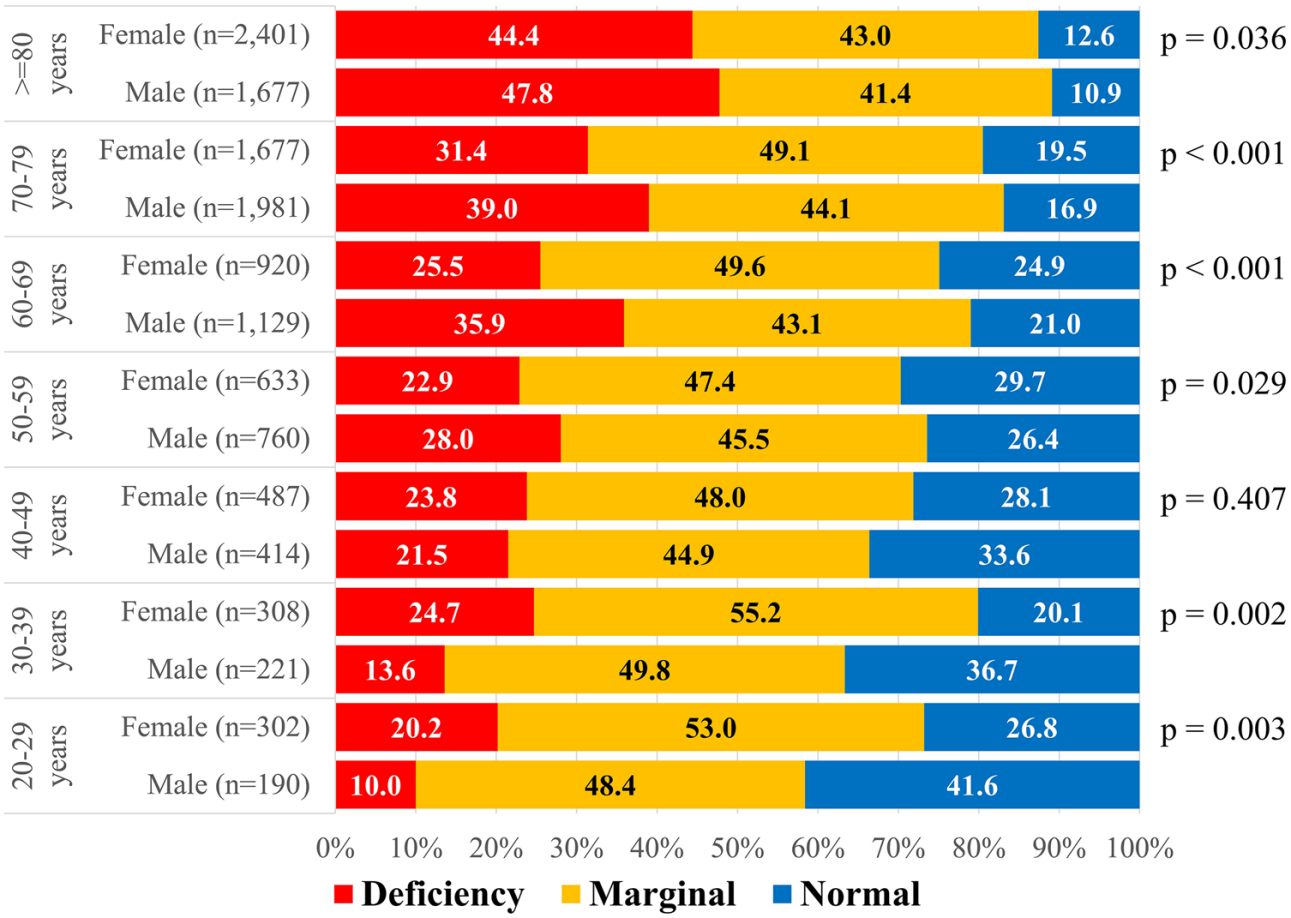
Summary of Serum Zinc Level by Sex and Age. Serum Zinc Level: Deficiency, < 60 μg/dL; Marginal, ≥ 60 to < 80 μg/dL; Normal, ≥ 80 μg/dL. *p*-values indicated chi-square tests comparing male and female Deficiency proportions within each age group.

Figure 2 presents a summary of serum zinc levels by age and sex. The proportion of patients with zinc deficiency increased with age for both sexes. Among those in their 60 s, 35.9% of males and 25.5% of females had zinc deficiency (*p* < 0.001). For patients in their 70 s, 39.0% of males and 31.4% of females had zinc deficiency (*p* < 0.001), and for those 80 and older, 47.8% of males and 44.4% of females had zinc deficiency (*p* = 0.036). Overall, for those in their 50 s and older, males consistently had a higher proportion of zinc deficiency compared to females. However, more females in their 20 s and 30 s had zinc deficiency compared to men in the same age group (*p* < 0.05). Supplementary Table S3 shows the odds ratios of zinc deficiency by sex and age.

Table 3 displays serum zinc levels by comorbidity recorded within 60 days before serum zinc-measurement. Comorbidities with higher proportion of zinc deficiency, in descending order, were: (1) pneumonitis due to solids and liquids (66.5%); (2) decubitus ulcer and pressure area (60.4%); (3) muscle wasting and atrophy, not elsewhere classified (sarcopenia) (56.7%); (4) chronic kidney disease (51.2%); (5) injuries to the head (48.1%); (6) hypertensive diseases (46.9%); (7) fracture (45.6%); (8) heart failure (45.4%); (9) cerebrovascular diseases (45.4%), and; (10) atrial fibrillation and flutter (45.3%). The results of subgroup analyses by age group, sex, and inpatient/outpatient status for Table 3 are presented in Supplementary Tables S4, S5 and S6, respectively.

**Table 3:** Summary of Serum Zinc Level by Comorbidity.

	Serum zinc level			
	Overall	Deficiency	Marginal	Normal
	n	n (%)	n (%)	n (%)
Total patients	13,100	4557 (34.8)	5964 (45.5)	2579 (19.7)
Comorbidity (within 60 days before serum zinc-measurement day)				
Intestinal infectious diseases	639	281 (44.0)	233 (36.5)	125 (19.6)
Noninfective enteritis and colitis	135	49 (36.3)	55 (40.7)	31 (23.0)
Tuberculosis	283	124 (43.8)	120 (42.4)	39 (13.8)
Malignant neoplasms of digestive organs	2536	1009 (39.8)	1022 (40.3)	505 (19.9)
Nutritional anemias	4144	1380 (33.3)	1995 (48.1)	769 (18.6)
Diabetes mellitus	4224	1427 (33.8)	1974 (46.7)	823 (19.5)
Hypogonadism	88	28 (31.8)	44 (50.0)	16 (18.2)
Short stature	81	27 (33.3)	39 (48.1)	15 (18.5)
Hyperlipidemia	1252	491 (39.2)	526 (42.0)	235 (18.8)
Hypertensive diseases	2447	1147 (46.9)	923 (37.7)	377 (15.4)
Acute myocardial infarction	873	259 (29.7)	445 (51.0)	169 (19.4)
Atrial fibrillation and flutter	662	300 (45.3)	268 (40.5)	94 (14.2)
Heart failure	2365	1074 (45.4)	960 (40.6)	331 (14.0)
Cerebrovascular diseases	1331	604 (45.4)	524 (39.4)	203 (15.3)
Influenza and pneumonia	2088	838 (40.1)	921 (44.1)	329 (15.8)
COVID-19	3640	1554 (42.7)	1507 (41.4)	579 (15.9)
Pneumonitis due to solids and liquids	439	292 (66.5)	116 (26.4)	31 (7.1)
Stomatitis and related lesions	247	63 (25.5)	121 (49.0)	63 (25.5)
Liver disease	2657	934 (35.2)	1155 (43.5)	568 (21.4)
Dermatitis and eczema	645	238 (36.9)	276 (42.8)	131 (20.3)
Alopecia areata	76	7 (9.2)	36 (47.4)	33 (43.4)
Decubitus ulcer and pressure area	265	160 (60.4)	83 (31.3)	22 (8.3)
Muscle wasting and atrophy, not elsewhere classified (Sarcopenia)	1166	661 (56.7)	371 (31.8)	134 (11.5)
Osteoporosis	823	349 (42.4)	339 (41.2)	135 (16.4)
Chronic kidney disease	1321	676 (51.2)	492 (37.2)	153 (11.6)
Disturbances of smell and taste	492	83 (16.9)	260 (52.8)	149 (30.3)
Anorexia	296	115 (38.9)	123 (41.6)	58 (19.6)
Injuries to the head	185	89 (48.1)	69 (37.3)	27 (14.6)
Fracture	834	380 (45.6)	322 (38.6)	132 (15.8)

Serum Zinc Level: Deficiency, < 60 μg/dL; Marginal, ≥ 60 to < 80 μg/dL; Normal, ≥ 80 μg/dL.

Table 4 displays serum zinc levels by medication recorded within 60 days before the zinc-measurement. Medications with higher proportion of zinc deficiency, in descending order, were: (1) spironolactone (58.4%); (2) furosemide (53.7%); (3) thyroid hormones (51.7%); (4) antianemic preparations (51.1%); (5) systemic antibacterials (50.4%); (6) anti-Parkinson agents (47.0%); (7) antithrombotic agents (45.8%); (8) antipsychotics (44.4%); (9) proton pump inhibitors (43.5%), and; (10) anxiolytics (43.2%). The results of subgroup analyses by age group, sex, and inpatient/outpatient status for Table 4 are presented in Supplementary Tables S7, S8 and S9, respectively.

**Table 4:** Summary of Serum Zinc Level by Medication.

	Serum zinc level			
	Overall	Deficiency	Marginal	Normal
	n	n (%)	n (%)	n (%)
Total patients	13,100	4557 (34.8)	5964 (45.5)	2579 (19.7)
Medication (within 60 days before serum zinc-measurement day)				
Antihyperglycemics	2191	793 (36.2)	972 (44.4)	426 (19.4)
Antihypertensive agents	5053	2165 (42.8)	2105 (41.7)	783 (15.5)
Spironolactone	818	478 (58.4)	257 (31.4)	83 (10.1)
Furosemide	2081	1117 (53.7)	741 (35.6)	223 (10.7)
ACE inhibitors	324	127 (39.2)	148 (45.7)	49 (15.1)
Angiotensin II receptor blockers	1862	693 (37.2)	826 (44.4)	343 (18.4)
Antihyperlipidemics	2148	634 (29.5)	1059 (49.3)	455 (21.2)
Statins	1861	558 (30.0)	924 (49.7)	379 (20.4)
Antithrombotic agents	3859	1767 (45.8)	1512 (39.2)	580 (15.0)
H2 blockers	930	352 (37.8)	406 (43.7)	172 (18.5)
Proton pump inhibitors	4273	1860 (43.5)	1697 (39.7)	716 (16.8)
Antianemic preparations	1935	989 (51.1)	730 (37.7)	216 (11.2)
Corticosteroids	1125	482 (42.8)	474 (42.1)	169 (15.0)
Thyroid hormones	410	212 (51.7)	141 (34.4)	57 (13.9)
Systemic antibacterials	3981	2006 (50.4)	1420 (35.7)	555 (13.9)
Drugs for treatment of bone diseases	484	164 (33.9)	240 (49.6)	80 (16.5)
Anti-Parkinson agents	215	101 (47.0)	91 (42.3)	23 (10.7)
Antipsychotics	1097	487 (44.4)	424 (38.7)	186 (17.0)
Anxiolytics	2442	1055 (43.2)	998 (40.9)	389 (15.9)

Serum Zinc Level: Deficiency, < 60 μg/dL; Marginal, ≥ 60 to < 80 μg/dL; Normal, ≥ 80 μg/dL.

*ACE* angiotensin-converting enzyme.

Supplementary Fig. S1 summarizes laboratory parameters recorded within 60 days before the zinc-measurement by serum zinc levels, and Supplementary Fig. S2 illustrates the correlation between these laboratory parameters and serum zinc concentration. Spearman's rank correlation coefficients for each variable with an absolute value of 0.3 or more were as follows: albumin (0.53); hemoglobin (0.42); CRP (− 0.38), and; total protein (0.36). In addition to the correlations described, Table S10 presents cross-table classifying hemoglobin levels into ‘Low’ and ‘Normal’, while concurrently categorizing serum zinc levels into ‘Deficiency’ and ‘Marginal or Normal.’ In Table S10, it was observed that the group with ‘Low’ hemoglobin levels had a significantly higher proportion of individuals with ‘Deficiency’ in Serum Zinc Levels compared to the group with ‘Normal’ hemoglobin levels (49.3% vs. 16.8%, *p* < 0.001).

Table 5 shows the association of comorbidity with zinc deficiency. The unadjusted and adjusted odds ratios of comorbidities for zinc deficiency were presented. In addition to univariate analysis, odds ratios adjusted for age group and sex were shown. The following comorbidities had the highest adjusted odds ratios, in descending order: (1) pneumonitis due to solids and liquids (2.959); (2) decubitus ulcer and pressure area (2.403), (3) muscle wasting and atrophy, not elsewhere classified (sarcopenia) (2.217); (4) COVID-19 (1.889); (5) chronic kidney disease (1.835); (6) intestinal infectious diseases (1.733); (7) hypertensive diseases (1.566); (8) injuries to the head (1.541); (9) influenza and pneumonia (1.490), and; (10) tuberculosis (1.480). As a result of sensitivity analysis for covariates, Supplementary Table S11 shows the demographic and clinical characteristics of inpatients/outpatients, and Supplementary Table S12 presents the adjusted odds ratios from a sensitivity analysis where inpatient/outpatient status is added to age group and sex as covariates, along with the unadjusted analysis for inpatients only and outpatients only.

**Table 5:** Unadjusted and Adjusted Odds Ratio for Zinc Deficiency with Comorbidities.

Zinc deficiency (< 60 μg/dL)	Unadjusted univariate analysis			Adjusted analysis^†^ by age group and sex		
	Odds ratio*	95% confidence interval	*p*-value	Odds ratio*	95% Confidence interval	*p*-value
Comorbidity (within 60 days before serum zinc-measurement day)						
Intestinal infectious diseases	1.502	(1.280, 1.764)	< .001	1.733	(1.468, 2.045)	< .001
Noninfective enteritis and colitis	1.070	(0.752, 1.522)	0.709	1.309	(0.910, 1.883)	0.147
Tuberculosis	1.475	(1.163, 1.871)	0.001	1.480	(1.160, 1.888)	0.002
Malignant neoplasms of digestive organs	1.307	(1.195, 1.429)	< .001	1.202	(1.097, 1.318)	< .001
Nutritional anemias	0.908	(0.840, 0.982)	0.015	0.929	(0.858, 1.006)	0.069
Diabetes mellitus	0.937	(0.867, 1.012)	0.096	0.917	(0.847, 0.992)	0.031
Hypogonadism	0.874	(0.557, 1.371)	0.558	0.960	(0.606, 1.522)	0.864
Short stature	0.938	(0.590, 1.490)	0.785	0.996	(0.620, 1.600)	0.986
Hyperlipidemia	1.235	(1.096, 1.392)	< .001	1.133	(1.003, 1.281)	0.044
Hypertensive diseases	1.874	(1.714, 2.049)	< .001	1.566	(1.429, 1.717)	< .001
Acute myocardial infarction	0.778	(0.670, 0.904)	0.001	0.868	(0.744, 1.013)	0.073
Atrial fibrillation and flutter	1.593	(1.361, 1.864)	< .001	1.215	(1.035, 1.427)	0.017
Heart failure	1.732	(1.582, 1.896)	< .001	1.470	(1.339, 1.613)	< .001
Cerebrovascular diseases	1.643	(1.465, 1.842)	< .001	1.376	(1.223, 1.547)	< .001
Influenza and pneumonia	1.315	(1.194, 1.447)	< .001	1.490	(1.348, 1.648)	< .001
COVID-19	1.602	(1.481, 1.733)	< .001	1.889	(1.738, 2.054)	< .001
Pneumonitis due to solids and liquids	3.910	(3.196, 4.784)	< .001	2.959	(2.410, 3.634)	< .001
Stomatitis and related lesions	0.637	(0.477, 0.850)	0.002	0.710	(0.529, 0.952)	0.022
Liver disease	1.020	(0.933, 1.116)	0.657	1.242	(1.131, 1.364)	< .001
Dermatitis and eczema	1.102	(0.935, 1.298)	0.248	1.115	(0.943, 1.318)	0.202
Alopecia areata	0.190	(0.087, 0.412)	< .001	0.306	(0.139, 0.673)	0.003
Decubitus ulcer and pressure area	2.922	(2.278, 3.748)	< .001	2.403	(1.866, 3.094)	< .001
Muscle wasting and atrophy, not elsewhere classified (Sarcopenia)	2.699	(2.389, 3.050)	< .001	2.217	(1.957, 2.512)	< .001
Osteoporosis	1.412	(1.224, 1.629)	< .001	1.302	(1.123, 1.509)	< .001
Chronic kidney disease	2.133	(1.902, 2.392)	< .001	1.835	(1.632, 2.063)	< .001
Disturbances of smell and taste	0.369	(0.291, 0.468)	< .001	0.416	(0.327, 0.531)	< .001
Anorexia	1.196	(0.944, 1.515)	0.138	1.042	(0.819, 1.325)	0.740
Injuries to the head	1.753	(1.311, 2.344)	< .001	1.541	(1.144, 2.074)	0.004
Fracture	1.621	(1.407, 1.867)	< .001	1.330	(1.150, 1.539)	< .001

*For each comorbidity, individuals without the comorbidity were used as the reference group to compare with those with the comorbidity for the calculation of odds ratios.

^†^Adjusted Analysis: Comorbidity (with vs without), Age Group (categorized as 20 s, 30 s, 40 s, 50 s, 60 s, 70 s, 80 and above), and Sex were specified as covariates in logistic regression, with an interaction term included between Age Group and Sex.

Table 6 shows the association of medication with zinc deficiency. Data were analyzed in a manner similar to comorbidities. Adjusted odds ratios, in descending order, were as follows: (1) spironolactone (2.523); (2) systemic antibacterials (2.419); (3) furosemide (2.138); (4) antianemic preparations (2.027); (5) thyroid hormones (1.864); (6) antithrombotic agents (1.621); (7) proton pump inhibitors (1.553); (8) corticosteroids (1.504); (9) anti-Parkinson agents (1.494), and; (10) antihypertensive agents (1.467). Supplementary Table S13 presents the adjusted odds ratios from a sensitivity analysis in which inpatient/outpatient status is added to age group and sex as covariates, along with the unadjusted analysis for inpatients only and outpatients only.

**Table 6:** Unadjusted and Adjusted Odds Ratio for Zinc Deficiency by Medications Prescribed.

Zinc deficiency (< 60 μg/dL)	Unadjusted univariate analysis			Adjusted analysis^†^ by age group and sex		
	Odds ratio*	95% confidence interval	*p*-value	Odds ratio*	95% confidence interval	*p*-value
Medication (within 60 days before serum zinc-measurement day)						
Antihyperglycemics	1.077	(0.979, 1.185)	0.130	0.955	(0.865, 1.054)	0.356
Antihypertensive agents	1.772	(1.647, 1.907)	< .001	1.467	(1.358, 1.584)	< .001
Spironolactone	2.826	(2.447, 3.264)	< .001	2.523	(2.179, 2.922)	< .001
Furosemide	2.552	(2.321, 2.807)	< .001	2.138	(1.939, 2.357)	< .001
ACE inhibitors	1.215	(0.969, 1.523)	0.092	0.974	(0.775, 1.225)	0.822
Angiotensin II receptor blockers	1.131	(1.022, 1.252)	0.017	0.987	(0.889, 1.095)	0.806
Antihyperlipidemics	0.750	(0.679, 0.830)	< .001	0.664	(0.599, 0.736)	< .001
Statins	0.775	(0.697, 0.862)	< .001	0.685	(0.614, 0.764)	< .001
Antithrombotic agents	1.953	(1.808, 2.110)	< .001	1.621	(1.496, 1.757)	< .001
H2 blockers	1.154	(1.005, 1.324)	0.042	1.108	(0.962, 1.275)	0.154
Proton pump inhibitors	1.752	(1.625, 1.890)	< .001	1.553	(1.436, 1.678)	< .001
Antianemic preparations	2.226	(2.019, 2.454)	< .001	2.027	(1.835, 2.239)	< .001
Corticosteroids	1.453	(1.284, 1.645)	< .001	1.504	(1.325, 1.708)	< .001
Thyroid hormones	2.056	(1.688, 2.504)	< .001	1.864	(1.524, 2.280)	< .001
Systemic antibacterials	2.615	(2.421, 2.825)	< .001	2.419	(2.235, 2.617)	< .001
Drugs for treatment of bone diseases	0.960	(0.792, 1.162)	0.673	0.849	(0.698, 1.033)	0.102
Anti-Parkinson agents	1.676	(1.279, 2.196)	< .001	1.494	(1.134, 1.968)	0.004
Antipsychotics	1.556	(1.373, 1.763)	< .001	1.411	(1.242, 1.603)	< .001
Anxiolytics	1.554	(1.421, 1.700)	< .001	1.372	(1.251, 1.503)	< .001

*ACE* angiotensin-converting enzyme.

*For each medication, individuals without the medication were used as the reference group to compare with those with the medication for the calculation of odds ratios.

^†^Adjusted Analysis: Medication (with vs without), Age Group (categorized as 20 s, 30 s, 40 s, 50 s, 60 s, 70 s, 80 and above), and Sex were specified as covariates in logistic regression, with an interaction term included between Age Group and Sex.

## Discussion

This study represents the first large-scale cross-sectional analysis of serum zinc levels in Japan using a nationwide medical claims database. The findings reveal associations between older adults, male sex, inpatient, and specific comorbidities such as respiratory infections, decubitus ulcer and pressure area, sarcopenia, and chronic kidney disease. Additionally, the use of medications such as spironolactone, furosemide, thyroid hormones, systemic antibacterials, and corticosteroids, was found to be associated with zinc deficiency. The associations between zinc deficiency and these specific comorbidities and medications were observed even after adjustments for age group and sex were made in multivariate analyses. By examining serum zinc levels across a diverse patient population, this study has clarified critical elements associated with zinc deficiency.

The study found zinc deficiency in 34.8% of the patients overall (36.6% in males, 33.1% in females). Notably, an increasing trend with age, showing a significant rise in zinc deficiency in older age groups was discovered (Fig. 2). Within this context, the findings revealed an association between age and zinc deficiency in both sexes. This might be due to older individuals' tendency towards lower zinc intake through their regular food consumption^36^. Moreover, past research has highlighted a correlation between aging-related inflammation and decreased serum zinc concentrations^37,38^.

Although this study identified differences between males and females in the impact of aging on zinc deficiency, it was observed that the odds of zinc deficiency increase for both sexes once individuals reach the age of 65 and above (Table S3). A previous large-scale study of healthy adults in Japan found a correlation between aging and serum zinc concentrations in males only^28^. While the relationship between sex and zinc deficiency is controversial and varies across studies^39^, this large-scale study contributes valuable evidence within the Japanese context.

Moreover, this study found that zinc deficiency was associated with respiratory infections. In fact, it was observed that patients with COVID-19 exhibited a higher prevalence of zinc deficiency across all age groups (Table S4), with zinc deficiency observed in 42.7% of the patients. Several reports have pointed out an association between COVID-19 and serum zinc concentration^40–45^. Similarly, patients with pneumonitis due to solids and liquids, influenza and pneumonia, and tuberculosis were also observed to have a higher prevalence of zinc deficiency, across all age groups. In relation to infections, zinc deficiency was noted in 50.4% and 42.8% of patients prescribed systemic antibacterials and corticosteroids, respectively. Zinc is known to affect antiviral immune responses and regulate the respiratory immune response^46^. A randomized controlled trial has confirmed the benefits of zinc supplementation for COVID-19 patients^42^. Consequently, clinicians might consider routinely monitoring zinc concentrations in patients with respiratory infections to ensure timely and appropriate intervention.

The study found an association between zinc deficiency and both decubitus ulcer and pressure area (bedsores) and sarcopenia, irrespective of age group (Table S4). Initially focusing on bedsores, previous studies have reported lower serum zinc levels in hospitalized patients^47,48^, likely due to zinc’s role in vital wound healing processes, including cell proliferation, inflammatory resolution, the immune system response, and antioxidant activity^13^. The study also found correlations between serum zinc concentration and each of the following laboratory parameters: albumin, total protein, and CRP (ρ = 0.53, 0.36, − 0.38, respectively (Fig. S2). International guidelines even recommend zinc-inclusive nutritional supplementation for patients with stage II or higher bedsores^49^.

Shifting attention to sarcopenia, it is crucial to remember that 60% of the body’s zinc is located in muscle^50^, suggesting low zinc levels may exacerbate sarcopenia by intensifying oxidative stress, resulting in muscle mass reduction^51^. Zinc's influence on sarcopenia is further evidenced in patients with chronic liver diseases^52^ and in relation to physical activity levels in the elderly^53,54^. As bedsores and sarcopenia related to aging are becoming increasingly prevalent^55,56^, particularly in Japan's rapidly aging population, the number of affected patients is projected to rise. Recently, the importance of nutrition management in the treatment of bedsores has been emphasized with the establishment of multidisciplinary nutritional support teams in hospitals^48,57^. Consequently, measuring serum zinc levels in patients with bedsores or sarcopenia might be beneficial as part of this nutritional management strategy. Additionally, raising awareness among nurses and dietitians about the comorbidities and medications linked with zinc deficiency could be advantageous.

An association between chronic kidney disease (CKD) and zinc deficiency was observed in this study. This result aligns with multiple studies conducted in Japan that have also suggested a link between zinc deficiency and CKD^21,58^. Furthermore, the research not only points to an association between CKD and zinc deficiency but also suggests that zinc deficiency might be a contributing factor to the progression of CKD^21^. Given these findings, there is an increasing interest in randomized controlled trials that focus on zinc supplementation therapy for CKD patients^59^. Such trials could offer valuable insights into the potential role of zinc in managing and treating CKD, underscoring the significance of micronutrient balance for renal health.

The current study’s observations revealed that conditions commonly associated with zinc deficiency or hypozincemia, such as nutritional anemia, diabetes mellitus, stomatitis and related lesions, alopecia areata, and disturbances of smell and taste, did not show expected associations with low zinc levels. While no direct correlation was found with diagnosed nutritional anemia (comorbidity), the higher proportion of observed zinc deficiency in the group with low hemoglobin levels (see Table S10) suggested that mild conditions, such as undiagnosed nutritional anemia, might be frequently overlooked in clinical settings. This oversight could potentially contribute to the observed findings. The lack of data on participants’ dietary zinc intake and use of zinc supplements could also account for these findings. It is plausible that individuals with these conditions may have used zinc supplements, consequently normalizing their zinc levels. The discrepancy between this study’s findings and the established associations in existing literature warrants further scrutiny, highlighting the complex nature of interactions between zinc deficiency and various health conditions.

A higher prevalence of zinc deficiency was observed in patients prescribed thyroid hormones, spironolactone, and furosemide, irrespective of age group (Table S7). Zinc is crucial for thyroid hormone function, managing specific enzyme activities, and aiding in hormone production critical for thyroid regulation^60^. This may explain the association between zinc deficiency and thyroid hormone usage observed in this study. Diuretics, on the other hand, are known to increase zinc excretion in patients with liver cirrhosis^61^, possibly contributing to the high prevalence of zinc deficiency among diuretic users. A previous study found a higher proportion of chronic kidney disease patients prescribed diuretics in the low-zinc group than in the high-zinc group^21^. Given the common prescription of thyroid hormones and diuretics, particularly among older individuals in Japan^62^, routine monitoring of serum zinc levels appears advisable.

This study has several limitations due to the nature of the MDV database that should be considered in subsequent investigations. First, the generalizability of the findings is limited due to selection bias present from including only patients with existing serum zinc concentration records from acute care hospitals. Second, the study's cross-sectional and observational nature means causal relationships cannot be established, and confounding lifestyle variables, including dietary zinc intake, smoking, and exercise habits, cannot be ruled out. Furthermore, although patients prescribed zinc-containing medication were excluded, some of the patients included may still be using zinc supplements or medications containing zinc prescribed at non-acute care hospitals and clinics, which are not captured in the MDV database. Another limitation is data completeness, with a high percentage of missing data for variables like weight and BMI, affecting the findings’ representativeness.

In the sensitivity analysis performed, the addition of inpatient/outpatient status as a covariate, along with age group and sex, to the multivariate analysis, significantly altered the adjusted odds ratios compared to the crude odds ratios, complicating the interpretation (Tables S12 and S13). Careful examination found that inpatients and outpatients exhibited differences in key demographic and clinical characteristics including age, prevalence of comorbidity, and medication usage (Table S11). Additionally, notable variations in crude odds ratios were observed for some comorbidities and medications, as outlined in Tables S12 and S13, when analyzing inpatient and outpatient groups separately. These findings imply a potential influence based on the patient’s inpatient or outpatient status. While the categorization of inpatient or outpatient status provides valuable contextual insights, it also introduces complex details regarding the patient's age, comorbidities, and medications. Considering the interdependencies between these variables, adding inpatient/outpatient status as a covariate in our multivariate analysis may affect the reliability and interpretability of the results concerning serum zinc levels. This complexity indicates that straightforward multivariate analysis might not be readily applicable, possibly due to the presence of diverse and unmeasured confounding variables and the complex interrelationships between covariates within the studied population. Identifying and selecting the most appropriate variables for inclusion in such analyses can be particularly difficult, as it requires careful consideration of their potential impact on study outcomes^63^. The use of large-scale real-world data in this study potentially exacerbates these challenges, rendering the interpretation of the analysis more nuanced and intricate.

Despite these limitations, the study provides a foundation for future research. Future investigations should aim to minimize selection bias and address issues related to missing data, while controlling for unmeasured confounders, particularly with respect to key variables such as weight and BMI. A longitudinal study design could facilitate establishing causation and tracking longitudinal changes in serum zinc levels over time. The inclusion of variables such as lifestyle, dietary habits, and zinc intake levels will provide a more comprehensive understanding of zinc deficiency. Additionally, future research should explore underlying mechanisms, dose–response relationships, and the effects of medication duration on serum zinc levels, to further validate associations with zinc deficiency. Taking into account the outcomes of the sensitivity analysis, future research may benefit from subgroup assessments based on key variables such as inpatient/outpatient status. Employing these thorough approaches may enable subsequent studies to provide more conclusive insights into the complex relationships between various factors, including medications, and zinc status.

## Conclusion

This cross-sectional study, using a nationwide Japanese claims database, identified several variables associated with zinc deficiency. These variables included being an older adult, male sex, inpatient status, and having comorbidities such as respiratory infections, decubitus ulcer and pressure area, sarcopenia, and chronic kidney disease. Additionally, the use of certain medications, namely spironolactone, furosemide, thyroid hormones, systemic antibacterials, and corticosteroids, was also found to be associated with zinc deficiency. These findings offer crucial guidance on which patients should be evaluated for serum zinc levels in clinical practice. This, in turn, can contribute to the early identification of zinc-deficient patients, facilitating timely and appropriate interventions and potentially improving outcomes in patient care. Moreover, advocating for zinc measurement in a broader population is crucial for data accumulation and research advancement. Future studies should prioritize investigating the mechanisms underlying these associations and monitoring changes in serum zinc levels to enhance the understanding of zinc deficiency.

### Supplementary Information

Supplementary Information.

## Data Availability

The data that support the findings of this study are available from Medical Data Vision Co., Ltd., but restrictions apply to the availability of these data, which were used under license for the current study, and so are not publicly available. Data are, however, available from the corresponding author, Nobuyuki Fukui, upon reasonable request and with permission of Medical Data Vision Co., Ltd.
