# Supplementary material for: Demographic and clinical characteristics of patients with zinc deficiency: analysis of a nationwide Japanese medical claims database
Source: Sci Rep. 2024 Feb 2;14:2791. doi: 10.1038/s41598-024-53202-0 (PMC10837122; doi:10.1038/s41598-024-53202-0)
Supplement: Supplementary file 1 — Supplementary Information. [file 41598_2024_53202_MOESM1_ESM.docx]

**Supplementary Materials**

# Demographic and clinical characteristics of patients with zinc deficiency: Analysis of a nationwide Japanese medical claims database

**Hirohide Yokokawa^1^, Yusuke Morita^2^, Izumi Hamada^2^, Yuji Ohta^2^, Nobuyuki Fukui^3, 4^*, Nao Makino^3, 4^, Emi Ohata^3, 4^, Toshio Naito^1^**

^1^ Department of General Medicine, Juntendo University Faculty of Medicine, Tokyo, Japan

^2^ Department of Data Science, Nobelpharma Co. Ltd, Tokyo, Japan

^3^ Department of Academic Services, 4DIN Ltd., Tokyo, Japan.

^4^ Center for Promotion of Data Science, Juntendo University Graduate School of Medicine, Tokyo, Japan

## *Corresponding author

Nobuyuki Fukui

#805 Shinbashiekimae Bldg.1 2-20-15 Shinbashi Minato-ku, Tokyo, 105-0004, JapanTel: +81-3-5537-6866

E-mail: nfukui@4din.com

Table S1. Definitions of Diseases by ICD 10 code

| Disease | ICD 10 Code |
| --- | --- |
| Intestinal infectious diseases | A00 - A09 |
| Noninfective enteritis and colitis | K50 - K52 |
| Tuberculosis | A15 - A19 |
| Malignant neoplasms of digestive organs | C15 - C26 |
| Nutritional anemias | D50 - D53 |
| Diabetes mellitus | E10 - E14 |
| Hypogonadism | E23.0; E23.1; E28.39; E29.1; E29.8; E29.9 |
| Short stature | E23.0; E34.3; P05.1 |
| Hyperlipidemia | E78.0 - E78.5 |
| Hypertensive diseases | I10 - I15 |
| Acute myocardial infarction | I21 |
| Atrial fibrillation and flutter | I48 |
| Heart failure | I50; I11.0 |
| Cerebrovascular diseases | I60 - I69 |
| Influenza and pneumonia | J09 - J18 |
| COVID-19 | U07.1; U07.2 |
| Pneumonitis due to solids and liquids | J69 |
| Stomatitis and related lesions | K12 |
| Liver disease | B15 - B19; K70 - K77 |
| Dermatitis and eczema | L20 - L30 |
| Alopecia areata | L63 |
| Decubitus ulcer and pressure area | L89 |
| Muscle wasting and atrophy, not elsewhere classified (Sarcopenia) | M62.5 |
| Osteoporosis | M80 - M82 |
| Chronic kidney disease | N18 - N19 |
| Disturbances of smell and taste | R43 |
| Anorexia | R63.0 |
| Injuries to the head | S00 - S09 |
| Fracture | S02; S12; S22; S32; S42; S52; S62; S72; S82; S92; T02; T08; T10; T12; T14.2; M80; M84.3; M84.4; M90.7; M96.6 |

ICD-10: International Classification of Diseases, 10th Revision.

Table S2. Definitions of concomitant medication by ATC code (EphMRA).

| Class/Name | ATC code  (EphMRA) |
| --- | --- |
| Antihyperglycemics | A10N1, A10N3, A10N9, A10P1, A10P5,  A10J1, A10K1, A10K2, A10K3, A10M1,  A10M9, A10C1, A10C2, A10C3, A10C5,  A10C9, A10L, A10H, A10D, A10S |
| Antihypertensive agents | C09D1, C09D3, C11A1, C02B2, C02A2,  C03A1, C03A2, C03A3, C03A7, C03A9,  C09A, C09C, C07A, C08A, C02D, C02C,  C09X, |
| Spironolactone | C03A1 |
| Furosemide | C03A2 |
| ACE inhibitors | C09A0 |
| Angiotensin II receptor blockers | C09C0 |
| Antihyperlipidemics (including statins)  (Lipid modifying agents) | C10A1, C11A1, C10A3, C10A4,  C10A9, C10C, C10B |
| Statins (only) | C10A1 |
| Antithrombotic agents | B01 |
| H2 blockers | A02B1 |
| Proton pump inhibitors | A02B2 |
| Antianemic preparations | B03 |
| Corticosteroids | H02A2 |
| Thyroid hormones | H03A0 |
| Systemic antibacterials | J01 |
| Drugs for treatment of bone diseases | M05B3 |
| Anti-Parkinson agents | N04A0 |
| Antipsychotics | N05A |
| Anxiolytics | N05B |

ATC: anatomical therapeutic chemical, ACE: angiotensin-converting enzyme, EphMRA: European Pharmaceutical Market Research Association.

Table S3. Unadjusted Odds Ratios for Zinc Deficiency with Age and Sex

|  | Overall (n=13,100) | | | Male (n=6,372) | | | Female (n=6,728) | | |
| --- | --- | --- | --- | --- | --- | --- | --- | --- | --- |
| Zinc Deficiency  (< 60 μg/dL) | Odds Ratio | 95%CI | *p*-value | Odds Ratio | 95%CI | *p*-value | Odds Ratio | 95%CI | *p*-value |
| **Age (years)  (on serum zinc-measurement day)^*^** | | | | | | | | | |
| Years/10 | 1.301 | (1.270, 1.332) | <.001 | 1.358 | (1.309, 1.409) | <.001 | 1.267 | (1.227, 1.307) | <.001 |
|  |  |  |  |  |  |  |  |  |  |
| **Age group  (on serum zinc-measurement day)^†^** | | | | | | | | | |
| 30-39years vs 20-29years | 1.290 | (0.937, 1.778) | 0.119 | 1.414 | (0.768, 2.603) | 0.267 | 1.294 | (0.883, 1.897) | 0.186 |
| 40-49years vs 20-29years | 1.517 | (1.140, 2.018) | 0.004 | 2.465 | (1.452, 4.182) | <.001 | 1.235 | (0.871, 1.753) | 0.236 |
| 50-59years vs 20-29years | 1.781 | (1.363, 2.329) | <.001 | 3.505 | (2.126, 5.776) | <.001 | 1.174 | (0.838, 1.644) | 0.350 |
| 60-69years vs 20-29years | 2.339 | (1.809, 3.025) | <.001 | 5.035 | (3.086, 8.212) | <.001 | 1.355 | (0.987, 1.862) | 0.061 |
| 70-79years vs 20-29years | 2.839 | (2.214, 3.641) | <.001 | 5.759 | (3.555, 9.330) | <.001 | 1.811 | (1.342, 2.442) | <.001 |
| >=80years vs 20-29years | 4.353 | (3.399, 5.574) | <.001 | 8.229 | (5.074, 13.347) | <.001 | 3.160 | (2.359, 4.233) | <.001 |
|  |  |  |  |  |  |  |  |  |  |
| **Age group  (with varied age cutoffs)^‡^** | | | | | | | | | |
| 40-64years vs 20-39years | 1.621 | (1.357, 1.936) | <.001 | 3.019 | (2.199, 4.143) | <.001 | 1.077 | (0.861, 1.348) | 0.517 |
| 65-79years vs 20-39years | 2.349 | (1.982, 2.783) | <.001 | 4.519 | (3.320, 6.151) | <.001 | 1.482 | (1.201, 1.830) | <.001 |
| >=80years vs 20-39years | 3.794 | (3.200, 4.500) | <.001 | 6.749 | (4.934, 9.232) | <.001 | 2.762 | (2.246, 3.395) | <.001 |
|  |  |  |  |  |  |  |  |  |  |

* Age was treated as a continuous variable, allowing for the estimation of changes in odds ratios per 10-year increase.

† Individuals aged 20 - 29 years were used as the control group to compare with other age groups.

‡ Individuals aged 20 - 39 years were used as the control group to compare with other age groups.

CI: Confidence Interval.

Figure S1. Summary of Laboratory Test by Serum Zinc Level

| 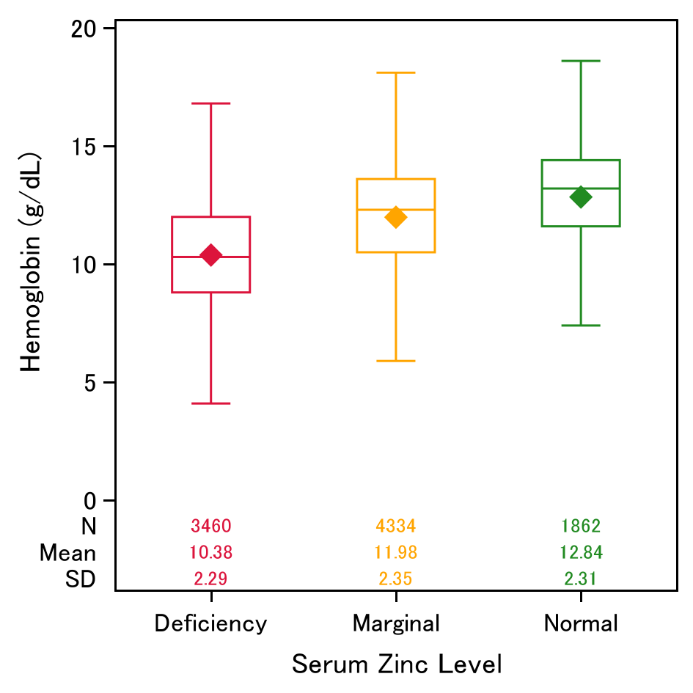 | 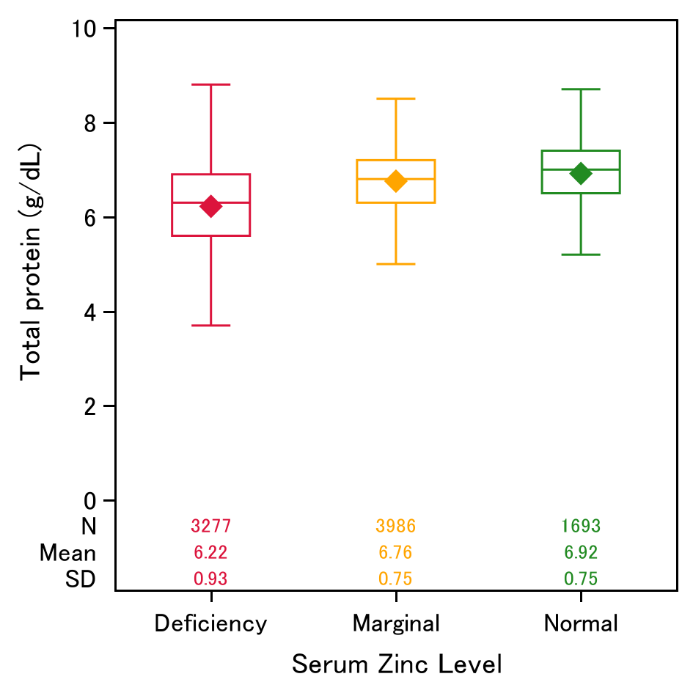 |
| --- | --- |
| 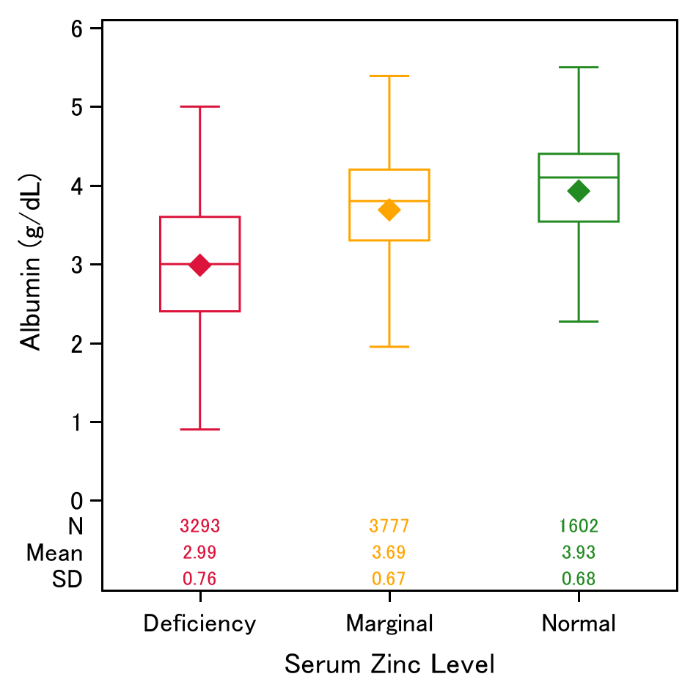 | 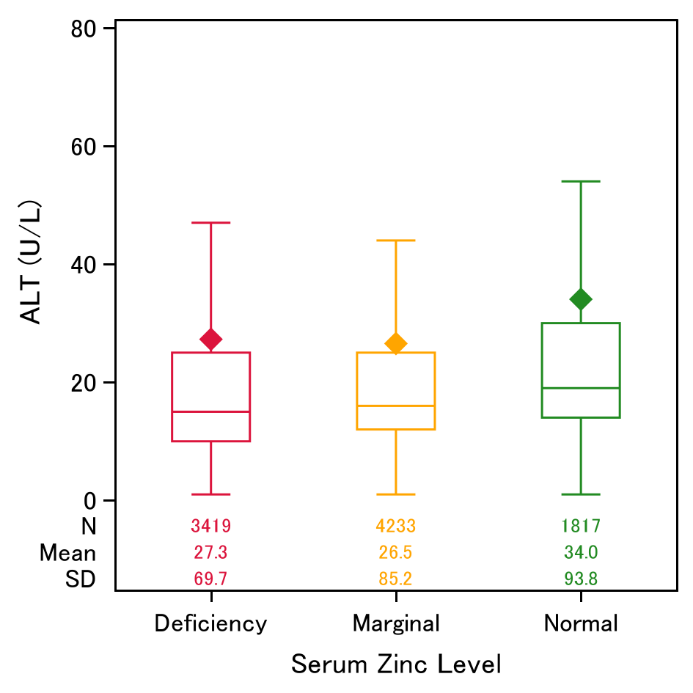 |
| 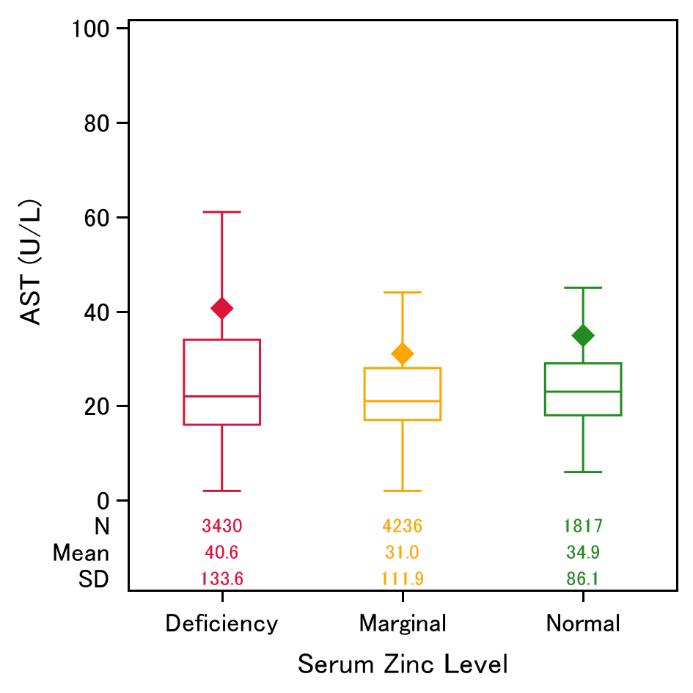 | 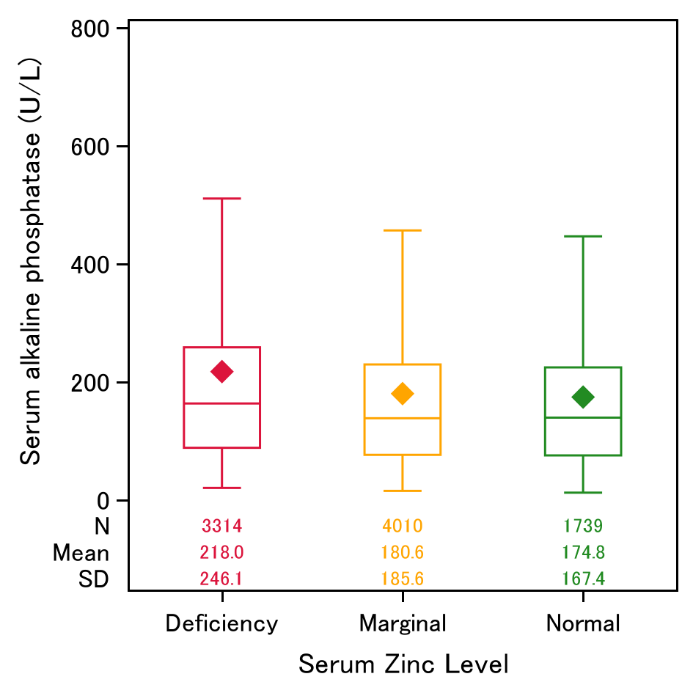 |

Serum Zinc Level: Deficiency, <60 μg/dL; Marginal, ≥60 to <80 μg/dL; Normal, ≥80 μg/dL.

Figure S1. Summary of Laboratory Test by Serum Zinc Level (cont.)

| 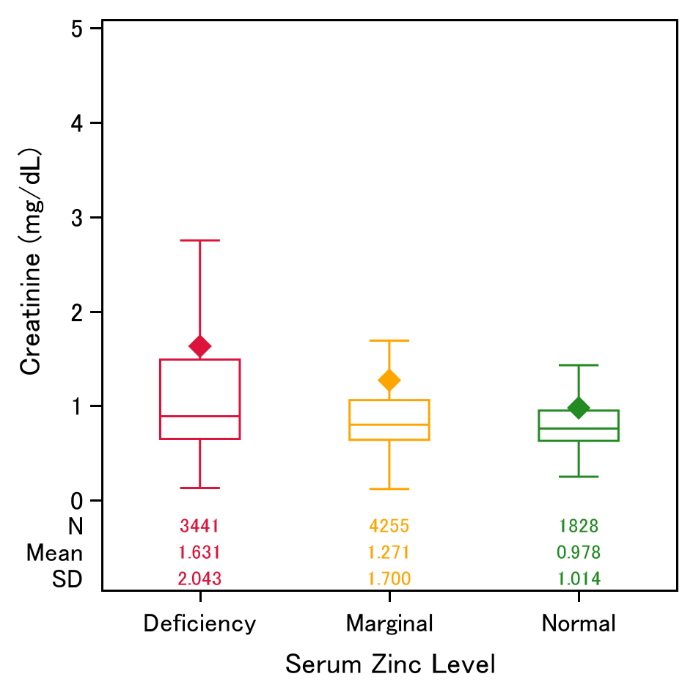 | 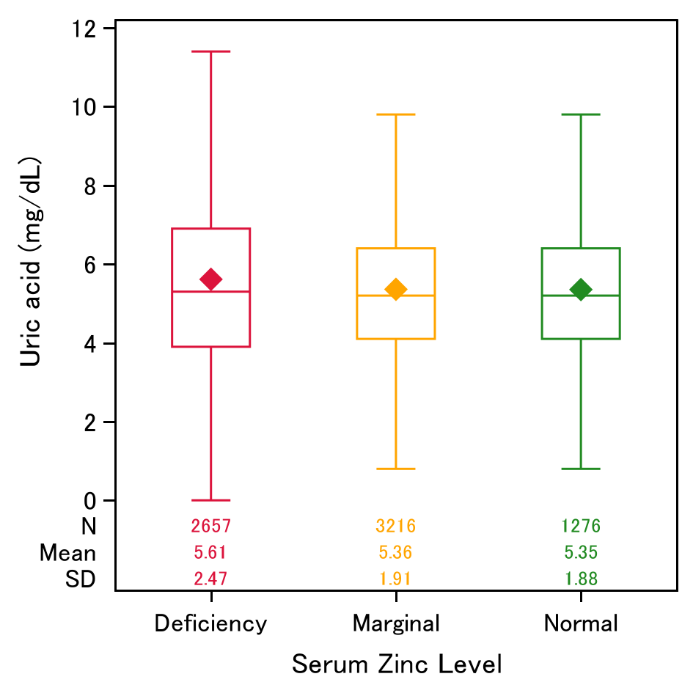 |
| --- | --- |
| 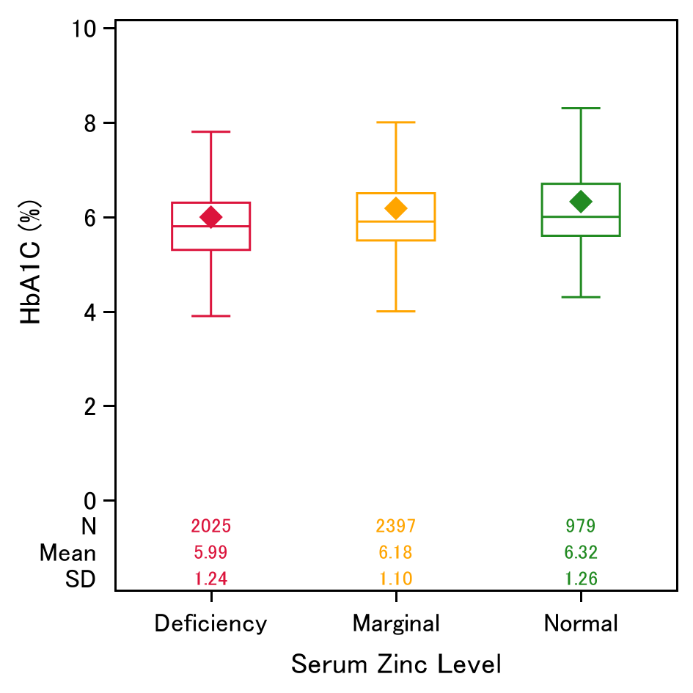 | 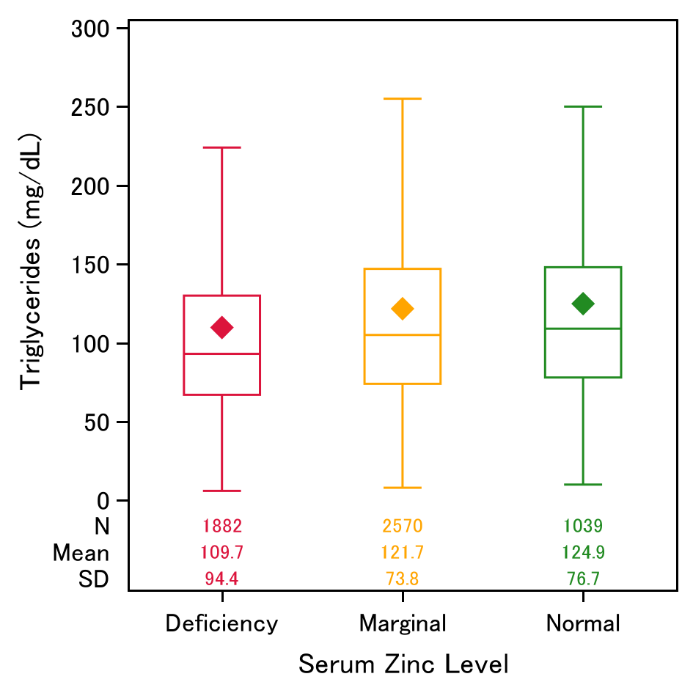 |
| 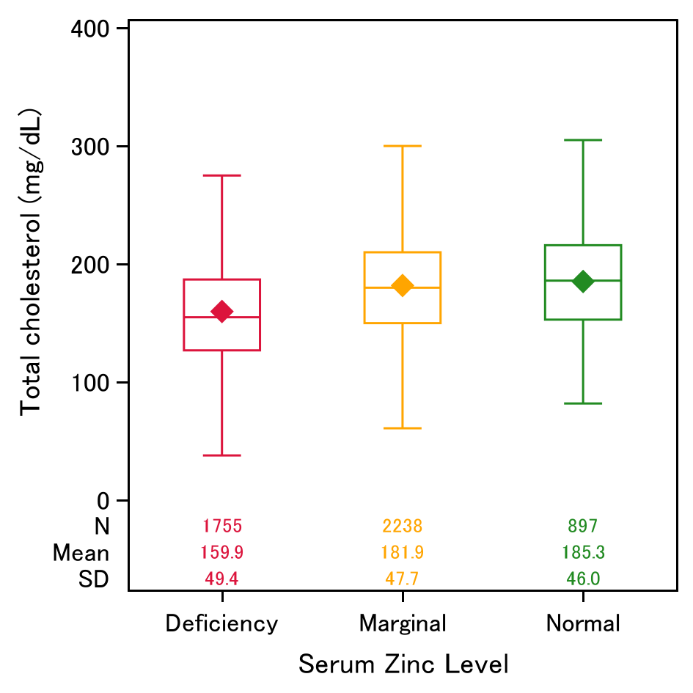 | 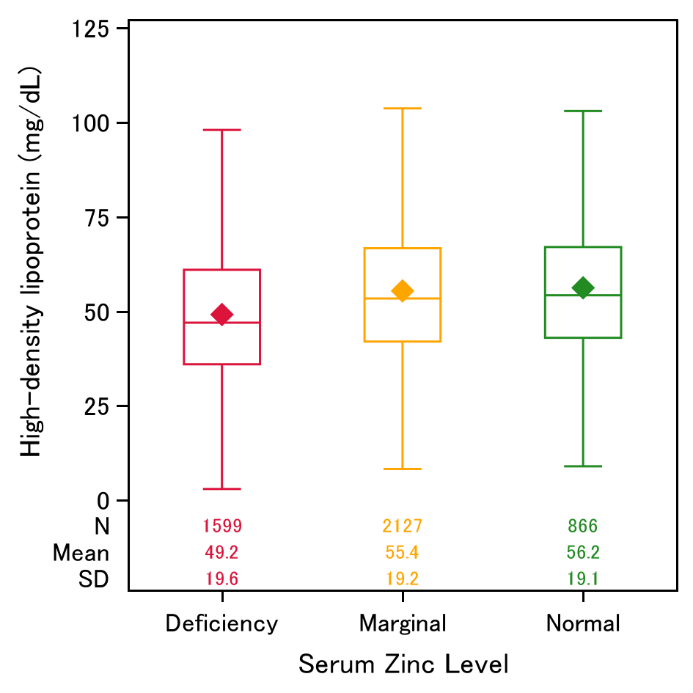 |

Serum Zinc Level: Deficiency, <60 μg/dL; Marginal, ≥60 to <80 μg/dL; Normal, ≥80 μg/dL.

Figure S1. Summary of Laboratory Test by Serum Zinc Level (cont.)

| 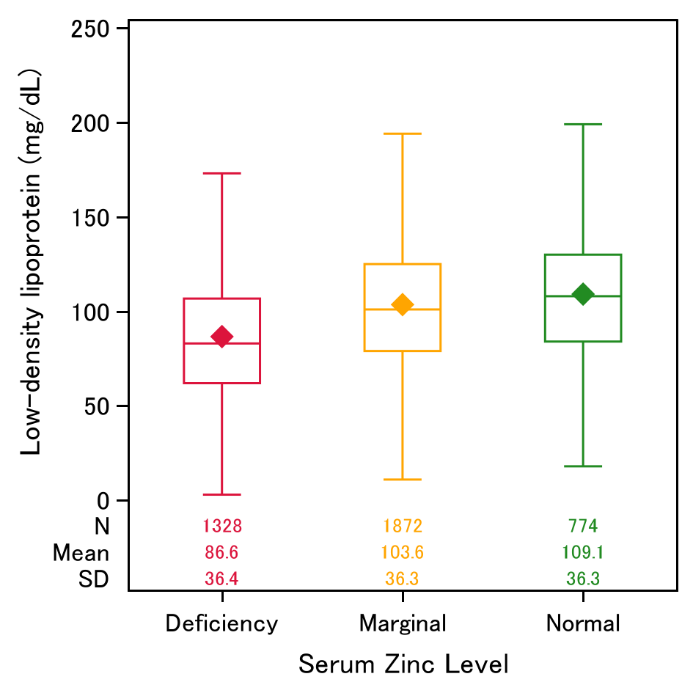 | 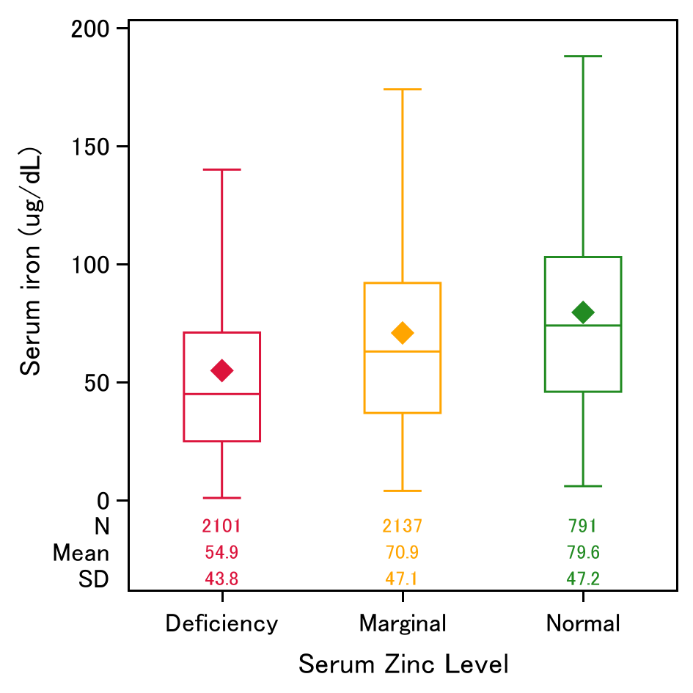 |
| --- | --- |
| 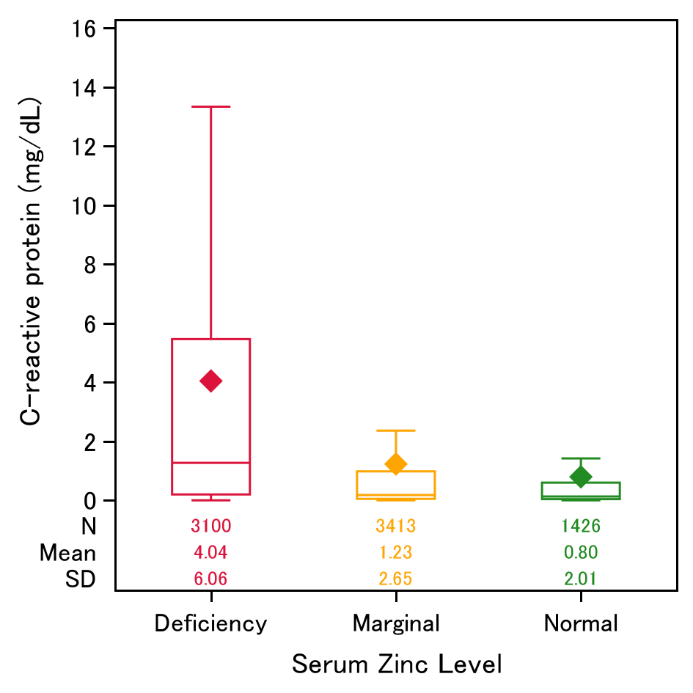 | 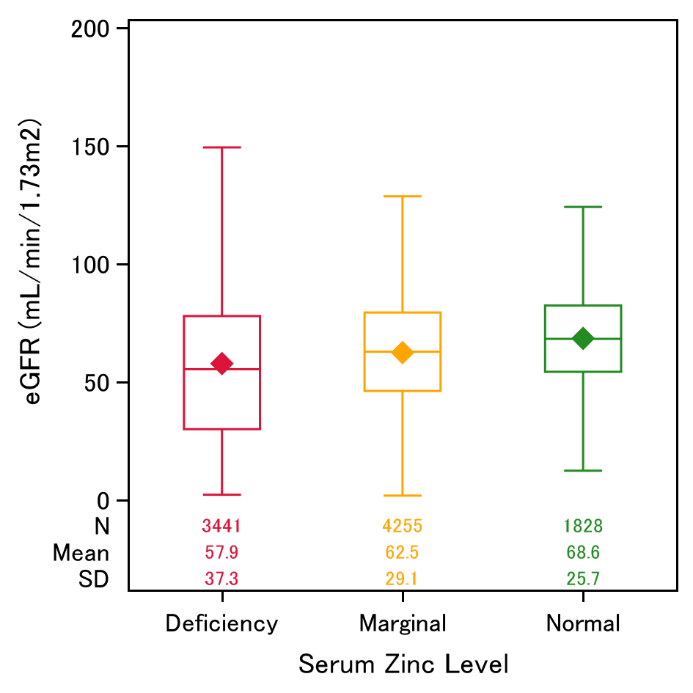 |
| 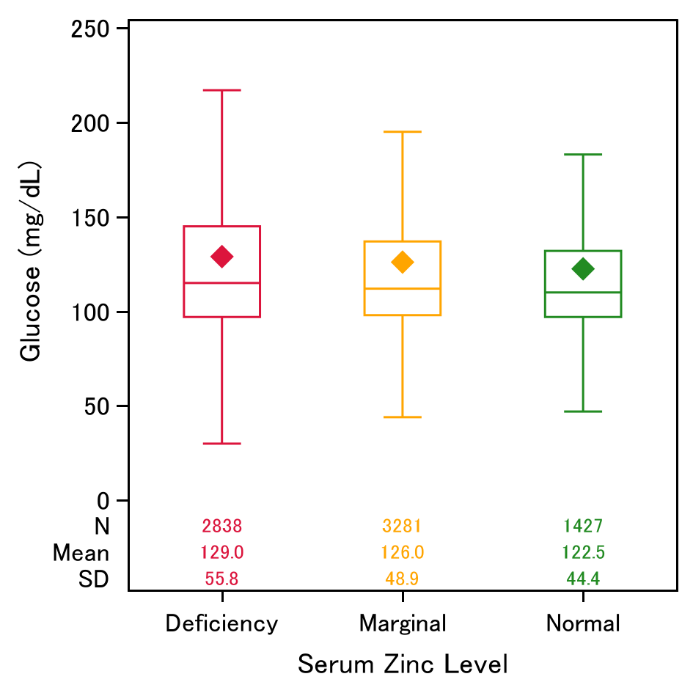 | 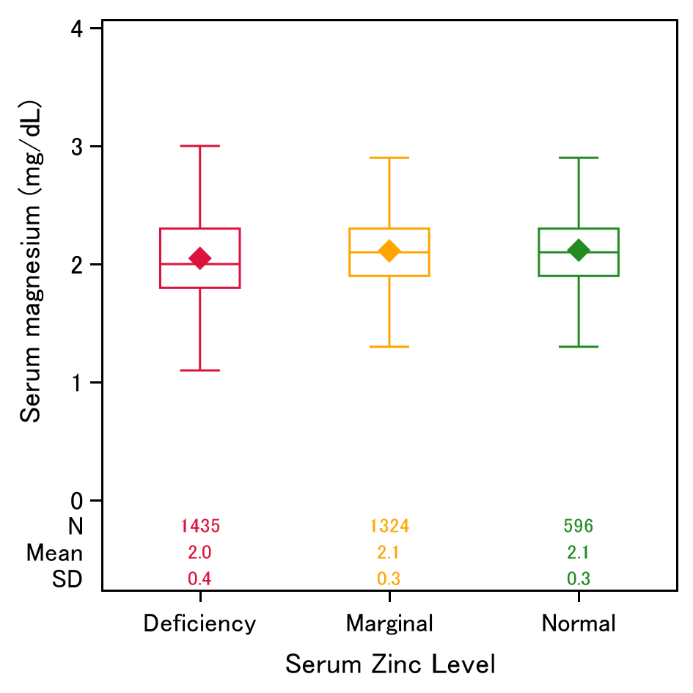 |

Serum Zinc Level: Deficiency, <60 μg/dL; Marginal, ≥60 to <80 μg/dL; Normal, ≥80 μg/dL.

Figure S2. Correlation of Laboratory Test with Serum Zinc Concentration


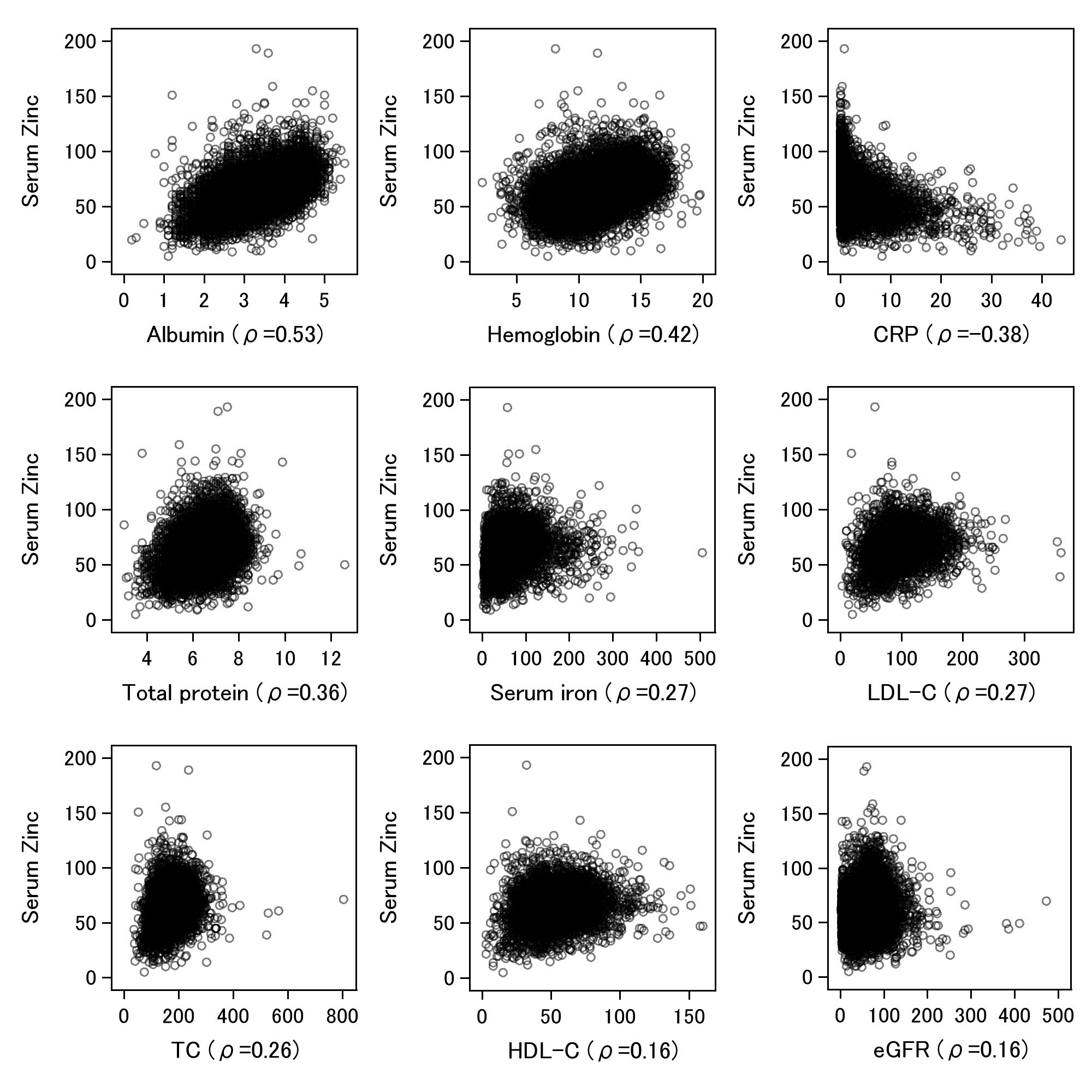


ρ : spearman’s rank coefficient of correlation.

Selected 9 laboratory test items in order of absolute value of spearman’s rank coefficient of correlation.

CRP: C-reactive protein, TC: Total cholesterol, HDL-C: High-density lipoprotein cholesterol, LDL-C: Low-density lipoprotein cholesterol.

Table S4. Summary of Serum Zinc Level by Comorbidity and Age Group

| Age Group | 20–39 years | | | | 40–64 years | | | | 65–79 years | | | |
| --- | --- | --- | --- | --- | --- | --- | --- | --- | --- | --- | --- | --- |
| Serum Zinc Level | Overall | Deficiency | Marginal | Normal | Overall | Deficiency | Marginal | Normal | Overall | Deficiency | Marginal | Normal |
|  | n | n (%) | n (%) | n (%) | n | n (%) | n (%) | n (%) | n | n (%) | n (%) | n (%) |
| **Total Patients** | 1,021 | 186(18.2) | 532(52.1) | 303(29.7) | 3,133 | 831(26.5) | 1,447(46.2) | 855(27.3) | 4,868 | 1,672(34.3) | 2,259(46.4) | 937(19.2) |
| **Comorbidity (within 60 days before serum zinc-measurement day)** | | | | | | | | | | | | |
| Intestinal infectious diseases | 94 | 30(31.9) | 43(45.7) | 21(22.3) | 176 | 64(36.4) | 74(42.0) | 38(21.6) | 222 | 100(45.0) | 79(35.6) | 43(19.4) |
| Noninfective enteritis and colitis | 22 | 2(9.1) | 9(40.9) | 11(50.0) | 45 | 14(31.1) | 21(46.7) | 10(22.2) | 44 | 21(47.7) | 17(38.6) | 6(13.6) |
| Tuberculosis | 19 | 2(10.5) | 12(63.2) | 5(26.3) | 64 | 21(32.8) | 30(46.9) | 13(20.3) | 113 | 50(44.2) | 49(43.4) | 14(12.4) |
| Malignant neoplasms of digestive organs | 51 | 10(19.6) | 22(43.1) | 19(37.3) | 537 | 176(32.8) | 229(42.6) | 132(24.6) | 1,168 | 463(39.6) | 464(39.7) | 241(20.6) |
| Nutritional anemias | 360 | 58(16.1) | 193(53.6) | 109(30.3) | 1,070 | 284(26.5) | 509(47.6) | 277(25.9) | 1,462 | 486(33.2) | 722(49.4) | 254(17.4) |
| Diabetes mellitus | 233 | 34(14.6) | 125(53.6) | 74(31.8) | 1,074 | 297(27.7) | 505(47.0) | 272(25.3) | 1,687 | 555(32.9) | 790(46.8) | 342(20.3) |
| Hypogonadism | 11 | 0(0.0) | 9(81.8) | 2(18.2) | 27 | 9(33.3) | 12(44.4) | 6(22.2) | 32 | 14(43.8) | 11(34.4) | 7(21.9) |
| Short stature | 10 | 0(0.0) | 8(80.0) | 2(20.0) | 21 | 8(38.1) | 8(38.1) | 5(23.8) | 32 | 14(43.8) | 11(34.4) | 7(21.9) |
| Hyperlipidemia | 41 | 10(24.4) | 20(48.8) | 11(26.8) | 252 | 78(31.0) | 111(44.0) | 63(25.0) | 488 | 181(37.1) | 208(42.6) | 99(20.3) |
| Hypertensive diseases | 25 | 11(44.0) | 7(28.0) | 7(28.0) | 379 | 147(38.8) | 152(40.1) | 80(21.1) | 920 | 423(46.0) | 337(36.6) | 160(17.4) |
| Acute myocardial infarction | 149 | 15(10.1) | 81(54.4) | 53(35.6) | 241 | 68(28.2) | 125(51.9) | 48(19.9) | 257 | 86(33.5) | 127(49.4) | 44(17.1) |
| Atrial fibrillation and flutter | 3 | 1(33.3) | 1(33.3) | 1(33.3) | 47 | 25(53.2) | 16(34.0) | 6(12.8) | 238 | 92(38.7) | 103(43.3) | 43(18.1) |
| Heart failure | 58 | 12(20.7) | 33(56.9) | 13(22.4) | 397 | 152(38.3) | 167(42.1) | 78(19.6) | 842 | 345(41.0) | 362(43.0) | 135(16.0) |
| Cerebrovascular diseases | 29 | 6(20.7) | 17(58.6) | 6(20.7) | 194 | 69(35.6) | 75(38.7) | 50(25.8) | 477 | 205(43.0) | 197(41.3) | 75(15.7) |
| Influenza and pneumonia | 380 | 79(20.8) | 196(51.6) | 105(27.6) | 476 | 161(33.8) | 232(48.7) | 83(17.4) | 613 | 271(44.2) | 263(42.9) | 79(12.9) |
| COVID-19 | 609 | 136(22.3) | 313(51.4) | 160(26.3) | 897 | 310(34.6) | 419(46.7) | 168(18.7) | 1,049 | 482(45.9) | 410(39.1) | 157(15.0) |
| Pneumonitis due to solids and liquids | 2 | 1(50.0) | 1(50.0) | 0(0.0) | 38 | 23(60.5) | 11(28.9) | 4(10.5) | 106 | 70(66.0) | 26(24.5) | 10(9.4) |
| Stomatitis and related lesions | 21 | 1(4.8) | 12(57.1) | 8(38.1) | 73 | 19(26.0) | 28(38.4) | 26(35.6) | 110 | 32(29.1) | 59(53.6) | 19(17.3) |
| Liver disease | 418 | 92(22.0) | 220(52.6) | 106(25.4) | 839 | 267(31.8) | 351(41.8) | 221(26.3) | 890 | 337(37.9) | 370(41.6) | 183(20.6) |
| Dermatitis and eczema | 44 | 4(9.1) | 24(54.5) | 16(36.4) | 169 | 49(29.0) | 76(45.0) | 44(26.0) | 252 | 99(39.3) | 108(42.9) | 45(17.9) |
| Alopecia areata | 30 | 0(0.0) | 18(60.0) | 12(40.0) | 39 | 6(15.4) | 12(30.8) | 21(53.8) | 3 | 0(0.0) | 3(100.0) | 0(0.0) |
| Decubitus ulcer and pressure area | 2 | 1(50.0) | 0(0.0) | 1(50.0) | 36 | 20(55.6) | 12(33.3) | 4(11.1) | 77 | 46(59.7) | 25(32.5) | 6(7.8) |
| Muscle wasting and atrophy, not elsewhere classified (Sarcopenia) | 8 | 4(50.0) | 3(37.5) | 1(12.5) | 145 | 85(58.6) | 40(27.6) | 20(13.8) | 400 | 215(53.8) | 133(33.3) | 52(13.0) |
| Osteoporosis | 16 | 7(43.8) | 8(50.0) | 1(6.3) | 155 | 53(34.2) | 71(45.8) | 31(20.0) | 271 | 103(38.0) | 117(43.2) | 51(18.8) |
| Chronic kidney disease | 20 | 6(30.0) | 8(40.0) | 6(30.0) | 219 | 87(39.7) | 96(43.8) | 36(16.4) | 523 | 273(52.2) | 194(37.1) | 56(10.7) |
| Disturbances of smell and taste | 52 | 6(11.5) | 28(53.8) | 18(34.6) | 160 | 14(8.8) | 73(45.6) | 73(45.6) | 193 | 38(19.7) | 109(56.5) | 46(23.8) |
| Anorexia | 9 | 3(33.3) | 4(44.4) | 2(22.2) | 40 | 15(37.5) | 14(35.0) | 11(27.5) | 117 | 37(31.6) | 51(43.6) | 29(24.8) |
| Injuries to the head | 8 | 2(25.0) | 2(25.0) | 4(50.0) | 28 | 10(35.7) | 13(46.4) | 5(17.9) | 60 | 28(46.7) | 24(40.0) | 8(13.3) |
| Fracture | 12 | 5(41.7) | 4(33.3) | 3(25.0) | 83 | 38(45.8) | 27(32.5) | 18(21.7) | 252 | 105(41.7) | 98(38.9) | 49(19.4) |

Serum Zinc Level: Deficiency, <60 μg/dL; Marginal, ≥60 to <80 μg/dL; Normal, ≥80 μg/dL.Table S4. Summary of Serum Zinc Level by Comorbidity and Age Group (cont.)

| Age Group | ≥ 80 years | | | |
| --- | --- | --- | --- | --- |
| Serum Zinc Level | Overall | Deficiency | Marginal | Normal |
|  | n | n (%) | n (%) | n (%) |
| **Total Patients** | 4,078 | 1,868(45.8) | 1,726(42.3) | 484(11.9) |
| **Comorbidity (within 60 days before serum zinc-measurement day)** |  |  |  |  |
| Intestinal infectious diseases | 147 | 87(59.2) | 37(25.2) | 23(15.6) |
| Noninfective enteritis and colitis | 24 | 12(50.0) | 8(33.3) | 4(16.7) |
| Tuberculosis | 87 | 51(58.6) | 29(33.3) | 7(8.0) |
| Malignant neoplasms of digestive organs | 780 | 360(46.2) | 307(39.4) | 113(14.5) |
| Nutritional anemias | 1,252 | 552(44.1) | 571(45.6) | 129(10.3) |
| Diabetes mellitus | 1,230 | 541(44.0) | 554(45.0) | 135(11.0) |
| Hypogonadism | 18 | 5(27.8) | 12(66.7) | 1(5.6) |
| Short stature | 18 | 5(27.8) | 12(66.7) | 1(5.6) |
| Hyperlipidemia | 471 | 222(47.1) | 187(39.7) | 62(13.2) |
| Hypertensive diseases | 1,123 | 566(50.4) | 427(38.0) | 130(11.6) |
| Acute myocardial infarction | 226 | 90(39.8) | 112(49.6) | 24(10.6) |
| Atrial fibrillation and flutter | 374 | 182(48.7) | 148(39.6) | 44(11.8) |
| Heart failure | 1,068 | 565(52.9) | 398(37.3) | 105(9.8) |
| Cerebrovascular diseases | 631 | 324(51.3) | 235(37.2) | 72(11.4) |
| Influenza and pneumonia | 619 | 327(52.8) | 230(37.2) | 62(10.0) |
| COVID-19 | 1,085 | 626(57.7) | 365(33.6) | 94(8.7) |
| Pneumonitis due to solids and liquids | 293 | 198(67.6) | 78(26.6) | 17(5.8) |
| Stomatitis and related lesions | 43 | 11(25.6) | 22(51.2) | 10(23.3) |
| Liver disease | 510 | 238(46.7) | 214(42.0) | 58(11.4) |
| Dermatitis and eczema | 180 | 86(47.8) | 68(37.8) | 26(14.4) |
| Alopecia areata | 4 | 1(25.0) | 3(75.0) | 0(0.0) |
| Decubitus ulcer and pressure area | 150 | 93(62.0) | 46(30.7) | 11(7.3) |
| Muscle wasting and atrophy, not elsewhere classified (Sarcopenia) | 613 | 357(58.2) | 195(31.8) | 61(10.0) |
| Osteoporosis | 381 | 186(48.8) | 143(37.5) | 52(13.6) |
| Chronic kidney disease | 559 | 310(55.5) | 194(34.7) | 55(9.8) |
| Disturbances of smell and taste | 87 | 25(28.7) | 50(57.5) | 12(13.8) |
| Anorexia | 130 | 60(46.2) | 54(41.5) | 16(12.3) |
| Injuries to the head | 89 | 49(55.1) | 30(33.7) | 10(11.2) |
| Fracture | 487 | 232(47.6) | 193(39.6) | 62(12.7) |

Serum Zinc Level: Deficiency, <60 μg/dL; Marginal, ≥60 to <80 μg/dL; Normal, ≥80 μg/dL.

Table S5. Summary of Serum Zinc Level by Comorbidity and Sex

| Sex | Male | | | | Female | | | |
| --- | --- | --- | --- | --- | --- | --- | --- | --- |
|  | | Serum Zinc Level | | |  | Serum Zinc Level | | |
|  | Overall | Deficiency | Marginal | Normal | Overall | Deficiency | Marginal | Normal |
|  | n | n (%) | n (%) | n (%) | N | n (%) | n (%) | n (%) |
| **Total Patients** | 6,372 | 2,330(36.6) | 2,789(43.8) | 1,253(19.7) | 6,728 | 2,227(33.1) | 3,175(47.2) | 1,326(19.7) |
| **Comorbidity (within 60 days before serum zinc-measurement day)** | | | | | | | | |
| Intestinal infectious diseases | 306 | 143(46.7) | 99(32.4) | 64(20.9) | 333 | 138(41.4) | 134(40.2) | 61(18.3) |
| Noninfective enteritis and colitis | 77 | 26(33.8) | 31(40.3) | 20(26.0) | 58 | 23(39.7) | 24(41.4) | 11(19.0) |
| Tuberculosis | 157 | 68(43.3) | 66(42.0) | 23(14.6) | 126 | 56(44.4) | 54(42.9) | 16(12.7) |
| Malignant neoplasms of digestive organs | 1,458 | 601(41.2) | 571(39.2) | 286(19.6) | 1,078 | 408(37.8) | 451(41.8) | 219(20.3) |
| Nutritional anemias | 2,003 | 707(35.3) | 922(46.0) | 374(18.7) | 2,141 | 673(31.4) | 1,073(50.1) | 395(18.4) |
| Diabetes mellitus | 2,269 | 812(35.8) | 1,021(45.0) | 436(19.2) | 1,955 | 615(31.5) | 953(48.7) | 387(19.8) |
| Hypogonadism | 47 | 18(38.3) | 21(44.7) | 8(17.0) | 41 | 10(24.4) | 23(56.1) | 8(19.5) |
| Short stature | 46 | 18(39.1) | 20(43.5) | 8(17.4) | 35 | 9(25.7) | 19(54.3) | 7(20.0) |
| Hyperlipidemia | 608 | 239(39.3) | 252(41.4) | 117(19.2) | 644 | 252(39.1) | 274(42.5) | 118(18.3) |
| Hypertensive diseases | 1,274 | 597(46.9) | 474(37.2) | 203(15.9) | 1,173 | 550(46.9) | 449(38.3) | 174(14.8) |
| Acute myocardial infarction | 482 | 141(29.3) | 241(50.0) | 100(20.7) | 391 | 118(30.2) | 204(52.2) | 69(17.6) |
| Atrial fibrillation and flutter | 368 | 167(45.4) | 144(39.1) | 57(15.5) | 294 | 133(45.2) | 124(42.2) | 37(12.6) |
| Heart failure | 1,276 | 582(45.6) | 517(40.5) | 177(13.9) | 1,089 | 492(45.2) | 443(40.7) | 154(14.1) |
| Cerebrovascular diseases | 725 | 336(46.3) | 274(37.8) | 115(15.9) | 606 | 268(44.2) | 250(41.3) | 88(14.5) |
| Influenza and pneumonia | 1,069 | 446(41.7) | 439(41.1) | 184(17.2) | 1,019 | 392(38.5) | 482(47.3) | 145(14.2) |
| COVID-19 | 1,796 | 752(41.9) | 736(41.0) | 308(17.1) | 1,844 | 802(43.5) | 771(41.8) | 271(14.7) |
| Pneumonitis due to solids and liquids | 272 | 188(69.1) | 69(25.4) | 15(5.5) | 167 | 104(62.3) | 47(28.1) | 16(9.6) |
| Stomatitis and related lesions | 103 | 26(25.2) | 49(47.6) | 28(27.2) | 144 | 37(25.7) | 72(50.0) | 35(24.3) |
| Liver disease | 1,356 | 513(37.8) | 550(40.6) | 293(21.6) | 1,301 | 421(32.4) | 605(46.5) | 275(21.1) |
| Dermatitis and eczema | 325 | 138(42.5) | 129(39.7) | 58(17.8) | 320 | 100(31.3) | 147(45.9) | 73(22.8) |
| Alopecia areata | 21 | 1(4.8) | 12(57.1) | 8(38.1) | 55 | 6(10.9) | 24(43.6) | 25(45.5) |
| Decubitus ulcer and pressure area | 126 | 74(58.7) | 40(31.7) | 12(9.5) | 139 | 86(61.9) | 43(30.9) | 10(7.2) |
| Muscle wasting and atrophy, not elsewhere classified (Sarcopenia) | 622 | 363(58.4) | 190(30.5) | 69(11.1) | 544 | 298(54.8) | 181(33.3) | 65(11.9) |
| Osteoporosis | 232 | 107(46.1) | 89(38.4) | 36(15.5) | 591 | 242(40.9) | 250(42.3) | 99(16.8) |
| Chronic kidney disease | 796 | 416(52.3) | 299(37.6) | 81(10.2) | 525 | 260(49.5) | 193(36.8) | 72(13.7) |
| Disturbances of smell and taste | 211 | 34(16.1) | 109(51.7) | 68(32.2) | 281 | 49(17.4) | 151(53.7) | 81(28.8) |
| Anorexia | 145 | 58(40.0) | 61(42.1) | 26(17.9) | 151 | 57(37.7) | 62(41.1) | 32(21.2) |
| Injuries to the head | 105 | 51(48.6) | 38(36.2) | 16(15.2) | 80 | 38(47.5) | 31(38.8) | 11(13.8) |
| Fracture | 276 | 129(46.7) | 105(38.0) | 42(15.2) | 558 | 251(45.0) | 217(38.9) | 90(16.1) |

Serum Zinc Level: Deficiency, <60 μg/dL; Marginal, ≥60 to <80 μg/dL; Normal, ≥80 μg/dL.

Table S6. Summary of Serum Zinc Level by Comorbidity and Inpatient/Outpatient

| Status | Inpatient | | | | Outpatient | | | |
| --- | --- | --- | --- | --- | --- | --- | --- | --- |
|  | | Serum Zinc Level | | |  | Serum Zinc Level | | |
|  | Overall | Deficiency | Marginal | Normal | Overall | Deficiency | Marginal | Normal |
|  | n | n (%) | n (%) | n (%) | N | n (%) | n (%) | n (%) |
| **Total Patients** | 5,614 | 2,822(50.3) | 1,995(35.5) | 797(14.2) | 7,420 | 1,713(23.1) | 3,938(53.1) | 1,769(23.8) |
| **Comorbidity (within 60 days before serum zinc-measurement day)** | | | | | | | | |
| Intestinal infectious diseases | 372 | 195(52.4) | 108(29.0) | 69(18.5) | 267 | 86(32.2) | 125(46.8) | 56(21.0) |
| Noninfective enteritis and colitis | 89 | 44(49.4) | 32(36.0) | 13(14.6) | 46 | 5(10.9) | 23(50.0) | 18(39.1) |
| Tuberculosis | 154 | 83(53.9) | 52(33.8) | 19(12.3) | 129 | 41(31.8) | 68(52.7) | 20(15.5) |
| Malignant neoplasms of digestive organs | 1,489 | 735(49.4) | 521(35.0) | 233(15.6) | 1,041 | 272(26.1) | 498(47.8) | 271(26.0) |
| Nutritional anemias | 1,387 | 709(51.1) | 517(37.3) | 161(11.6) | 2,750 | 669(24.3) | 1,475(53.6) | 606(22.0) |
| Diabetes mellitus | 2,029 | 902(44.5) | 806(39.7) | 321(15.8) | 2,189 | 522(23.8) | 1,166(53.3) | 501(22.9) |
| Hypogonadism | 38 | 17(44.7) | 16(42.1) | 5(13.2) | 48 | 10(20.8) | 27(56.3) | 11(22.9) |
| Short stature | 38 | 17(44.7) | 16(42.1) | 5(13.2) | 42 | 9(21.4) | 23(54.8) | 10(23.8) |
| Hyperlipidemia | 924 | 407(44.0) | 351(38.0) | 166(18.0) | 327 | 83(25.4) | 175(53.5) | 69(21.1) |
| Hypertensive diseases | 2,026 | 992(49.0) | 723(35.7) | 311(15.4) | 421 | 155(36.8) | 200(47.5) | 66(15.7) |
| Acute myocardial infarction | 425 | 161(37.9) | 189(44.5) | 75(17.6) | 447 | 97(21.7) | 256(57.3) | 94(21.0) |
| Atrial fibrillation and flutter | 582 | 268(46.0) | 235(40.4) | 79(13.6) | 80 | 32(40.0) | 33(41.3) | 15(18.8) |
| Heart failure | 1,579 | 832(52.7) | 568(36.0) | 179(11.3) | 782 | 240(30.7) | 390(49.9) | 152(19.4) |
| Cerebrovascular diseases | 975 | 500(51.3) | 339(34.8) | 136(13.9) | 356 | 104(29.2) | 185(52.0) | 67(18.8) |
| Influenza and pneumonia | 1,108 | 563(50.8) | 409(36.9) | 136(12.3) | 976 | 274(28.1) | 510(52.3) | 192(19.7) |
| COVID-19 | 2,299 | 1,187(51.6) | 807(35.1) | 305(13.3) | 1,335 | 366(27.4) | 695(52.1) | 274(20.5) |
| Pneumonitis due to solids and liquids | 425 | 284(66.8) | 110(25.9) | 31(7.3) | 14 | 8(57.1) | 6(42.9) | 0(0.0) |
| Stomatitis and related lesions | 88 | 36(40.9) | 36(40.9) | 16(18.2) | 158 | 26(16.5) | 85(53.8) | 47(29.7) |
| Liver disease | 1,081 | 550(50.9) | 354(32.7) | 177(16.4) | 1,572 | 382(24.3) | 799(50.8) | 391(24.9) |
| Dermatitis and eczema | 343 | 163(47.5) | 121(35.3) | 59(17.2) | 302 | 75(24.8) | 155(51.3) | 72(23.8) |
| Alopecia areata | 4 | 1(25.0) | 2(50.0) | 1(25.0) | 72 | 6(8.3) | 34(47.2) | 32(44.4) |
| Decubitus ulcer and pressure area | 249 | 151(60.6) | 79(31.7) | 19(7.6) | 16 | 9(56.3) | 4(25.0) | 3(18.8) |
| Muscle wasting and atrophy, not elsewhere classified (Sarcopenia) | 1,126 | 647(57.5) | 352(31.3) | 127(11.3) | 39 | 13(33.3) | 19(48.7) | 7(17.9) |
| Osteoporosis | 533 | 263(49.3) | 185(34.7) | 85(15.9) | 288 | 84(29.2) | 154(53.5) | 50(17.4) |
| Chronic kidney disease | 910 | 507(55.7) | 311(34.2) | 92(10.1) | 406 | 167(41.1) | 179(44.1) | 60(14.8) |
| Disturbances of smell and taste | 48 | 15(31.3) | 22(45.8) | 11(22.9) | 443 | 68(15.3) | 238(53.7) | 137(30.9) |
| Anorexia | 201 | 90(44.8) | 74(36.8) | 37(18.4) | 95 | 25(26.3) | 49(51.6) | 21(22.1) |
| Injuries to the head | 149 | 73(49.0) | 55(36.9) | 21(14.1) | 36 | 16(44.4) | 14(38.9) | 6(16.7) |
| Fracture | 706 | 334(47.3) | 262(37.1) | 110(15.6) | 128 | 46(35.9) | 60(46.9) | 22(17.2) |

Serum Zinc Level: Deficiency, <60 μg/dL; Marginal, ≥60 to <80 μg/dL; Normal, ≥80 μg/dL.

Table S7. Summary of Serum Zinc Level by Medication and Age Group

| Age Group | 20–39 years | | | | 40–64 years | | | | 65–79 years | | | |
| --- | --- | --- | --- | --- | --- | --- | --- | --- | --- | --- | --- | --- |
| Serum Zinc Level | Overall | Deficiency | Marginal | Normal | Overall | Deficiency | Marginal | Normal | Overall | Deficiency | Marginal | Normal |
|  | n | n (%) | n (%) | n (%) | n | n (%) | n (%) | n (%) | n | n (%) | n (%) | n (%) |
| **Total Patients** | 1,021 | 186(18.2) | 532(52.1) | 303(29.7) | 3,133 | 831(26.5) | 1,447(46.2) | 855(27.3) | 4,868 | 1,672(34.3) | 2,259(46.4) | 937(19.2) |
| **Medication (within 60 days before serum zinc-measurement day)** | | | | | | | | | | | | |
| Antihyperglycemics | 22 | 4(18.2) | 10(45.5) | 8(36.4) | 443 | 118(26.6) | 194(43.8) | 131(29.6) | 1,056 | 377(35.7) | 468(44.3) | 211(20.0) |
| Antihypertensive agents | 68 | 30(44.1) | 23(33.8) | 15(22.1) | 887 | 312(35.2) | 389(43.9) | 186(21.0) | 2,022 | 815(40.3) | 859(42.5) | 348(17.2) |
| Spironolactone | 14 | 10(71.4) | 3(21.4) | 1(7.1) | 149 | 94(63.1) | 39(26.2) | 16(10.7) | 310 | 175(56.5) | 92(29.7) | 43(13.9) |
| Furosemide | 32 | 18(56.3) | 9(28.1) | 5(15.6) | 307 | 165(53.7) | 99(32.2) | 43(14.0) | 735 | 389(52.9) | 263(35.8) | 83(11.3) |
| ACE inhibitors | 2 | 0(0.0) | 2(100.0) | 0(0.0) | 39 | 11(28.2) | 22(56.4) | 6(15.4) | 126 | 39(31.0) | 63(50.0) | 24(19.0) |
| Angiotensin II receptor blockers | 17 | 4(23.5) | 7(41.2) | 6(35.3) | 361 | 104(28.8) | 163(45.2) | 94(26.0) | 775 | 283(36.5) | 353(45.5) | 139(17.9) |
| Antihyperlipidemics | 15 | 2(13.3) | 7(46.7) | 6(40.0) | 407 | 79(19.4) | 199(48.9) | 129(31.7) | 997 | 281(28.2) | 501(50.3) | 215(21.6) |
| Statins | 13 | 2(15.4) | 6(46.2) | 5(38.5) | 343 | 67(19.5) | 171(49.9) | 105(30.6) | 865 | 250(28.9) | 433(50.1) | 182(21.0) |
| Antithrombotic agents | 57 | 21(36.8) | 20(35.1) | 16(28.1) | 591 | 239(40.4) | 236(39.9) | 116(19.6) | 1,560 | 676(43.3) | 626(40.1) | 258(16.5) |
| H2 blockers | 35 | 7(20.0) | 17(48.6) | 11(31.4) | 220 | 64(29.1) | 104(47.3) | 52(23.6) | 391 | 158(40.4) | 158(40.4) | 75(19.2) |
| Proton pump inhibitors | 90 | 25(27.8) | 40(44.4) | 25(27.8) | 848 | 316(37.3) | 325(38.3) | 207(24.4) | 1,788 | 744(41.6) | 739(41.3) | 305(17.1) |
| Antianemic preparations | 66 | 23(34.8) | 28(42.4) | 15(22.7) | 384 | 176(45.8) | 150(39.1) | 58(15.1) | 737 | 366(49.7) | 292(39.6) | 79(10.7) |
| Corticosteroids | 43 | 10(23.3) | 25(58.1) | 8(18.6) | 297 | 102(34.3) | 135(45.5) | 60(20.2) | 510 | 238(46.7) | 193(37.8) | 79(15.5) |
| Thyroid hormones | 11 | 2(18.2) | 7(63.6) | 2(18.2) | 59 | 30(50.8) | 19(32.2) | 10(16.9) | 159 | 82(51.6) | 44(27.7) | 33(20.8) |
| Systemic antibacterials | 212 | 71(33.5) | 104(49.1) | 37(17.5) | 751 | 326(43.4) | 289(38.5) | 136(18.1) | 1,509 | 733(48.6) | 543(36.0) | 233(15.4) |
| Drugs for treatment of bone diseases | 5 | 1(20.0) | 4(80.0) | 0(0.0) | 61 | 25(41.0) | 25(41.0) | 11(18.0) | 193 | 54(28.0) | 100(51.8) | 39(20.2) |
| Anti-Parkinson agents | 3 | 1(33.3) | 2(66.7) | 0(0.0) | 41 | 14(34.1) | 24(58.5) | 3(7.3) | 79 | 36(45.6) | 32(40.5) | 11(13.9) |
| Antipsychotics | 43 | 9(20.9) | 21(48.8) | 13(30.2) | 221 | 78(35.3) | 96(43.4) | 47(21.3) | 391 | 181(46.3) | 142(36.3) | 68(17.4) |
| Anxiolytics | 65 | 17(26.2) | 29(44.6) | 19(29.2) | 444 | 154(34.7) | 193(43.5) | 97(21.8) | 958 | 401(41.9) | 398(41.5) | 159(16.6) |

Serum Zinc Level: Deficiency, <60 μg/dL; Marginal, ≥60 to <80 μg/dL; Normal, ≥80 μg/dL.

ACE: angiotensin-converting enzyme.

Table S7. Summary of Serum Zinc Level by Medication and Age Group (cont.)

| Age Group | ≥ 80 years | | | |
| --- | --- | --- | --- | --- |
| Serum Zinc Level | Overall | Deficiency | Marginal | Normal |
|  | n | n (%) | n (%) | n (%) |
| **Total Patients** | 4,078 | 1,868(45.8) | 1,726(42.3) | 484(11.9) |
| **Medication (within 60 days before serum zinc-measurement day)** |  |  |  |  |
| Antihyperglycemics | 670 | 294(43.9) | 300(44.8) | 76(11.3) |
| Antihypertensive agents | 2,076 | 1,008(48.6) | 834(40.2) | 234(11.3) |
| Spironolactone | 345 | 199(57.7) | 123(35.7) | 23(6.7) |
| Furosemide | 1,007 | 545(54.1) | 370(36.7) | 92(9.1) |
| ACE inhibitors | 157 | 77(49.0) | 61(38.9) | 19(12.1) |
| Angiotensin II receptor blockers | 709 | 302(42.6) | 303(42.7) | 104(14.7) |
| Antihyperlipidemics | 729 | 272(37.3) | 352(48.3) | 105(14.4) |
| Statins | 640 | 239(37.3) | 314(49.1) | 87(13.6) |
| Antithrombotic agents | 1,651 | 831(50.3) | 630(38.2) | 190(11.5) |
| H2 blockers | 284 | 123(43.3) | 127(44.7) | 34(12.0) |
| Proton pump inhibitors | 1,547 | 775(50.1) | 593(38.3) | 179(11.6) |
| Antianemic preparations | 748 | 424(56.7) | 260(34.8) | 64(8.6) |
| Corticosteroids | 275 | 132(48.0) | 121(44.0) | 22(8.0) |
| Thyroid hormones | 181 | 98(54.1) | 71(39.2) | 12(6.6) |
| Systemic antibacterials | 1,509 | 876(58.1) | 484(32.1) | 149(9.9) |
| Drugs for treatment of bone diseases | 225 | 84(37.3) | 111(49.3) | 30(13.3) |
| Anti-Parkinson agents | 92 | 50(54.3) | 33(35.9) | 9(9.8) |
| Antipsychotics | 442 | 219(49.5) | 165(37.3) | 58(13.1) |
| Anxiolytics | 975 | 483(49.5) | 378(38.8) | 114(11.7) |

Serum Zinc Level: Deficiency, <60 μg/dL; Marginal, ≥60 to <80 μg/dL; Normal, ≥80 μg/dL.

ACE: angiotensin-converting enzyme.

Table S8. Summary of Serum Zinc Level by Medication and Sex

| Sex | Male | | | | Female | | | |
| --- | --- | --- | --- | --- | --- | --- | --- | --- |
|  | | Serum Zinc Level | | |  | Serum Zinc Level | | |
|  | Overall | Deficiency | Marginal | Normal | Overall | Deficiency | Marginal | Normal |
|  | n | n (%) | n (%) | n (%) | n | n (%) | n (%) | n (%) |
| **Total Patients** | 6,372 | 2,330(36.6) | 2,789(43.8) | 1,253(19.7) | 6,728 | 2,227(33.1) | 3,175(47.2) | 1,326(19.7) |
| **Medication (within 60 days before serum zinc-measurement day)** | | | | | | | | |
| Antihyperglycemics | 1,357 | 500(36.8) | 602(44.4) | 255(18.8) | 834 | 293(35.1) | 370(44.4) | 171(20.5) |
| Antihypertensive agents | 2,728 | 1,189(43.6) | 1,121(41.1) | 418(15.3) | 2,325 | 976(42.0) | 984(42.3) | 365(15.7) |
| Spironolactone | 453 | 280(61.8) | 129(28.5) | 44(9.7) | 365 | 198(54.2) | 128(35.1) | 39(10.7) |
| Furosemide | 1,158 | 626(54.1) | 420(36.3) | 112(9.7) | 923 | 491(53.2) | 321(34.8) | 111(12.0) |
| ACE inhibitors | 188 | 70(37.2) | 93(49.5) | 25(13.3) | 136 | 57(41.9) | 55(40.4) | 24(17.6) |
| Angiotensin II receptor blockers | 1,015 | 379(37.3) | 445(43.8) | 191(18.8) | 847 | 314(37.1) | 381(45.0) | 152(17.9) |
| Antihyperlipidemics | 1,090 | 326(29.9) | 538(49.4) | 226(20.7) | 1,058 | 308(29.1) | 521(49.2) | 229(21.6) |
| Statins | 938 | 289(30.8) | 470(50.1) | 179(19.1) | 923 | 269(29.1) | 454(49.2) | 200(21.7) |
| Antithrombotic agents | 2,135 | 979(45.9) | 831(38.9) | 325(15.2) | 1,724 | 788(45.7) | 681(39.5) | 255(14.8) |
| H2 blockers | 504 | 201(39.9) | 203(40.3) | 100(19.8) | 426 | 151(35.4) | 203(47.7) | 72(16.9) |
| Proton pump inhibitors | 2,324 | 1,032(44.4) | 904(38.9) | 388(16.7) | 1,949 | 828(42.5) | 793(40.7) | 328(16.8) |
| Antianemic preparations | 1,029 | 561(54.5) | 370(36.0) | 98(9.5) | 906 | 428(47.2) | 360(39.7) | 118(13.0) |
| Corticosteroids | 564 | 243(43.1) | 234(41.5) | 87(15.4) | 561 | 239(42.6) | 240(42.8) | 82(14.6) |
| Thyroid hormones | 178 | 99(55.6) | 55(30.9) | 24(13.5) | 232 | 113(48.7) | 86(37.1) | 33(14.2) |
| Systemic antibacterials | 2,104 | 1,045(49.7) | 748(35.6) | 311(14.8) | 1,877 | 961(51.2) | 672(35.8) | 244(13.0) |
| Drugs for treatment of bone diseases | 116 | 55(47.4) | 45(38.8) | 16(13.8) | 368 | 109(29.6) | 195(53.0) | 64(17.4) |
| Anti-Parkinson agents | 111 | 57(51.4) | 44(39.6) | 10(9.0) | 104 | 44(42.3) | 47(45.2) | 13(12.5) |
| Antipsychotics | 563 | 251(44.6) | 220(39.1) | 92(16.3) | 534 | 236(44.2) | 204(38.2) | 94(17.6) |
| Anxiolytics | 1,232 | 536(43.5) | 510(41.4) | 186(15.1) | 1,210 | 519(42.9) | 488(40.3) | 203(16.8) |

Serum Zinc Level: Deficiency, <60 μg/dL; Marginal, ≥60 to <80 μg/dL; Normal, ≥80 μg/dL.

ACE: angiotensin-converting enzyme

Table S9. Summary of Serum Zinc Level by Medication and Inpatient/Outpatient

| Status | Inpatient | | | | Outpatient | | | |
| --- | --- | --- | --- | --- | --- | --- | --- | --- |
|  | | Serum Zinc Level | | |  | Serum Zinc Level | | |
|  | Overall | Deficiency | Marginal | Normal | Overall | Deficiency | Marginal | Normal |
|  | n | n (%) | n (%) | n (%) | n | n (%) | n (%) | n (%) |
| **Total Patients** | 5,614 | 2,822(50.3) | 1,995(35.5) | 797(14.2) | 7,420 | 1,713(23.1) | 3,938(53.1) | 1,769(23.8) |
| **Medication (within 60 days before serum zinc-measurement day)** | | | | | | | | |
| Antihyperglycemics | 1,348 | 609(45.2) | 505(37.5) | 234(17.4) | 836 | 182(21.8) | 464(55.5) | 190(22.7) |
| Antihypertensive agents | 3,058 | 1,559(51.0) | 1,078(35.3) | 421(13.8) | 1,981 | 602(30.4) | 1,020(51.5) | 359(18.1) |
| Spironolactone | 623 | 372(59.7) | 185(29.7) | 66(10.6) | 195 | 106(54.4) | 72(36.9) | 17(8.7) |
| Furosemide | 1,552 | 880(56.7) | 503(32.4) | 169(10.9) | 528 | 237(44.9) | 237(44.9) | 54(10.2) |
| ACE inhibitors | 213 | 102(47.9) | 80(37.6) | 31(14.6) | 111 | 25(22.5) | 68(61.3) | 18(16.2) |
| Angiotensin II receptor blockers | 987 | 454(46.0) | 375(38.0) | 158(16.0) | 869 | 236(27.2) | 449(51.7) | 184(21.2) |
| Antihyperlipidemics | 985 | 414(42.0) | 407(41.3) | 164(16.6) | 1,151 | 216(18.8) | 646(56.1) | 289(25.1) |
| Statins | 869 | 372(42.8) | 362(41.7) | 135(15.5) | 980 | 182(18.6) | 556(56.7) | 242(24.7) |
| Antithrombotic agents | 2,839 | 1,397(49.2) | 1,015(35.8) | 427(15.0) | 1,013 | 366(36.1) | 494(48.8) | 153(15.1) |
| H2 blockers | 530 | 241(45.5) | 194(36.6) | 95(17.9) | 400 | 111(27.8) | 212(53.0) | 77(19.3) |
| Proton pump inhibitors | 2,749 | 1,394(50.7) | 942(34.3) | 413(15.0) | 1,511 | 461(30.5) | 749(49.6) | 301(19.9) |
| Antianemic preparations | 1,151 | 651(56.6) | 386(33.5) | 114(9.9) | 781 | 337(43.1) | 342(43.8) | 102(13.1) |
| Corticosteroids | 627 | 306(48.8) | 229(36.5) | 92(14.7) | 497 | 175(35.2) | 245(49.3) | 77(15.5) |
| Thyroid hormones | 271 | 166(61.3) | 73(26.9) | 32(11.8) | 138 | 46(33.3) | 67(48.6) | 25(18.1) |
| Systemic antibacterials | 3,007 | 1,649(54.8) | 938(31.2) | 420(14.0) | 971 | 356(36.7) | 480(49.4) | 135(13.9) |
| Drugs for treatment of bone diseases | 178 | 87(48.9) | 59(33.1) | 32(18.0) | 303 | 76(25.1) | 179(59.1) | 48(15.8) |
| Anti-Parkinson agents | 162 | 78(48.1) | 67(41.4) | 17(10.5) | 53 | 23(43.4) | 24(45.3) | 6(11.3) |
| Antipsychotics | 921 | 429(46.6) | 338(36.7) | 154(16.7) | 174 | 56(32.2) | 86(49.4) | 32(18.4) |
| Anxiolytics | 1,794 | 845(47.1) | 670(37.3) | 279(15.6) | 639 | 206(32.2) | 325(50.9) | 108(16.9) |

Serum Zinc Level: Deficiency, <60 μg/dL; Marginal, ≥60 to <80 μg/dL; Normal, ≥80 μg/dL.

ACE: angiotensin-converting enzyme

Table S10. Association of Hemoglobin Level and Serum Zinc Level

|  | | Serum Zinc Level^†^ | |  |
| --- | --- | --- | --- | --- |
| Sex | Hemoglobin^*^ Level | Deficiency n (%) | Marginal or Normal n (%) | p-value (chi-square) |
| Overall | Normal | 674 (16.8%) | 3,335 (83.2%) | <.001 |
|  | Low | 2,786 (49.3%) | 2,861 (50.7%) |  |
| Male | Normal | 276 (16.1%) | 1,440 (83.9%) | <.001 |
|  | Low | 1,486 (50.7%) | 1,445 (49.3%) |  |
| Female | Normal | 398 (17.4%) | 1,895 (82.6%) | <.001 |
|  | Low | 1,300 (47.9%) | 1,416 (52.1%) |  |

* Hemoglobin Level: Low (Male < 13 g/dL, Female < 12 g/dL), Normal (Male >= 13 g/dL, Female >= 12 g/dL).

† Serum Zinc Level: Deficiency, <60 μg/dL; Marginal, ≥60 to <80 μg/dL; Normal, ≥80 μg/dL.

Table S11. Summary of Demographic and Clinical Characteristics by Inpatient/Outpatient

|  | | Status | | |
| --- | --- | --- | --- | --- |
| Demographic/Clinical | Overall | Inpatient | Outpatient | missing |
| Characteristics | n | n (%) | n (%) | n (%) |
| **Total Patients** | | | | |
| - | 13,100 | 5,614(42.9) | 7,420(56.6) | 66(0.5) |
| **Serum Zinc** | | | | |
| Mean±SD (n) | 65.9±17.6 (n=13100) | 60.4±18.9 (n=5614) | 70.1±15.3 (n=7420) | 68.0±17.6 (n=66) |
| **Sex** | | | | |
| Male | 6,372 | 2,976(46.7) | 3,370(52.9) | 26(0.4) |
| Female | 6,728 | 2,638(39.2) | 4,050(60.2) | 40(0.6) |
| **Age (years)  (on serum zinc-measurement day)** | | | | |
| Mean±SD (n) | 69.0±17.1 (n=13100) | 73.8±14.8 (n=5614) | 65.3±17.8 (n=7420) | 70.5±13.6 (n=66) |
| **Age group  (on serum zinc-measurement day)** | | | | |
| 20-29 years | 492 | 80(16.3) | 411(83.5) | 1(0.2) |
| 30-39 years | 529 | 93(17.6) | 433(81.9) | 3(0.6) |
| 40-49 years | 901 | 248(27.5) | 652(72.4) | 1(0.1) |
| 50-59 years | 1,393 | 486(34.9) | 900(64.6) | 7(0.5) |
| 60-69 years | 2,049 | 799(39.0) | 1,239(60.5) | 11(0.5) |
| 70-79 years | 3,658 | 1,566(42.8) | 2,066(56.5) | 26(0.7) |
| >=80 years | 4,078 | 2,342(57.4) | 1,719(42.2) | 17(0.4) |
| **Weight(kg)** | | | | |
| Mean±SD (n) | 54.4±14.2 (n=4772) | 54.3±14.3 (n=4565) | 56.6±12.7 (n=204) | 57.7±23.5 (n=3) |
| **BMI(kg/m^2^)** | | | | |
| Mean±SD (n) | 21.7±4.5 (n=4676) | 21.7±4.5 (n=4470) | 22.1±4.0 (n=203) | 22.1±5.7 (n=3) |
| **BMI group** | | | | |
| < 25 | 3,750 | 3,581(95.5) | 167(4.5) | 2(0.1) |
| >=25 | 926 | 889(96.0) | 36(3.9) | 1(0.1) |
| Missing | 8,424 | 1,144(13.6) | 7,217(85.7) | 63(0.7) |
| **Smoking** | | | | |
| No | 2,897 | 2,772(95.7) | 122(4.2) | 3(0.1) |
| Yes | 1,723 | 1,638(95.1) | 85(4.9) | 0(0.0) |
| Missing | 8,480 | 1,204(14.2) | 7,213(85.1) | 63(0.7) |

Table S11. Summary of Demographic and Clinical Characteristics by Inpatient/Outpatient (cont.)

|  | | Status | | |
| --- | --- | --- | --- | --- |
| Demographic/Clinical | Overall | Inpatient | Outpatient | missing |
| Characteristics | n | n (%) | n (%) | n (%) |
| **Comorbidity  (within 60 days before serum zinc-measurement day)** | | | | |
| Intestinal infectious diseases | 639 | 372(58.2) | 267(41.8) | 0(0.0) |
| Noninfective enteritis and colitis | 135 | 89(65.9) | 46(34.1) | 0(0.0) |
| Tuberculosis | 283 | 154(54.4) | 129(45.6) | 0(0.0) |
| Malignant neoplasms of digestive organs | 2,536 | 1,489(58.7) | 1,041(41.0) | 6(0.2) |
| Nutritional anaemias | 4,144 | 1,387(33.5) | 2,750(66.4) | 7(0.2) |
| Diabetes mellitus | 4,224 | 2,029(48.0) | 2,189(51.8) | 6(0.1) |
| Hypogonadism | 88 | 38(43.2) | 48(54.5) | 2(2.3) |
| Short statue | 81 | 38(46.9) | 42(51.9) | 1(1.2) |
| Hyperlipidemia | 1,252 | 924(73.8) | 327(26.1) | 1(0.1) |
| Hypertensive diseases | 2,447 | 2,026(82.8) | 421(17.2) | 0(0.0) |
| Acute myocardial infarction | 873 | 425(48.7) | 447(51.2) | 1(0.1) |
| Atrial fibrillation and flutter | 662 | 582(87.9) | 80(12.1) | 0(0.0) |
| Heart failure | 2,365 | 1,579(66.8) | 782(33.1) | 4(0.2) |
| Cerebrovascular diseases | 1,331 | 975(73.3) | 356(26.7) | 0(0.0) |
| Influenza and pneumonia | 2,088 | 1,108(53.1) | 976(46.7) | 4(0.2) |
| COVID-19 | 3,640 | 2,299(63.2) | 1,335(36.7) | 6(0.2) |
| Pneumonitis due to solids and liquids | 439 | 425(96.8) | 14(3.2) | 0(0.0) |
| Stomatitis and related lesions | 247 | 88(35.6) | 158(64.0) | 1(0.4) |
| Liver disease | 2,657 | 1,081(40.7) | 1,572(59.2) | 4(0.2) |
| Dermatitis and eczema | 645 | 343(53.2) | 302(46.8) | 0(0.0) |
| Alopecia areata | 76 | 4(5.3) | 72(94.7) | 0(0.0) |
| Decubitus ulcer and pressure area | 265 | 249(94.0) | 16(6.0) | 0(0.0) |
| Muscle wasting and atrophy, not elsewhere classified (Sarcopenia) | 1,166 | 1,126(96.6) | 39(3.3) | 1(0.1) |
| Osteoporosis | 823 | 533(64.8) | 288(35.0) | 2(0.2) |
| Chronic kidney disease | 1,321 | 910(68.9) | 406(30.7) | 5(0.4) |
| Disturbances of smell and taste | 492 | 48(9.8) | 443(90.0) | 1(0.2) |
| Anorexia | 296 | 201(67.9) | 95(32.1) | 0(0.0) |
| Injuries to the head | 185 | 149(80.5) | 36(19.5) | 0(0.0) |
| Fracture | 834 | 706(84.7) | 128(15.3) | 0(0.0) |

Table S11. Summary of Demographic and Clinical Characteristics by Inpatient/Outpatient (cont.)

|  | | Status | | |
| --- | --- | --- | --- | --- |
| Demographic/Clinical | Overall | Inpatient | Outpatient | missing |
| Characteristics | n | n (%) | n (%) | n (%) |
| **Medication (within 60 days before serum zinc-measurement day)** | | | | |
| Antihyperglycemics | 2,191 | 1,348(61.5) | 836(38.2) | 7(0.3) |
| Antihypertensive agents | 5,053 | 3,058(60.5) | 1,981(39.2) | 14(0.3) |
| Spironolactone | 818 | 623(76.2) | 195(23.8) | 0(0.0) |
| Furosemide | 2,081 | 1,552(74.6) | 528(25.4) | 1(0.0) |
| ACE inhibitors | 324 | 213(65.7) | 111(34.3) | 0(0.0) |
| Angiotensin II receptor blockers | 1,862 | 987(53.0) | 869(46.7) | 6(0.3) |
| Antihyperlipidemics | 2,148 | 985(45.9) | 1,151(53.6) | 12(0.6) |
| Statins | 1,861 | 869(46.7) | 980(52.7) | 12(0.6) |
| Antithrombotic agents | 3,859 | 2,839(73.6) | 1,013(26.3) | 7(0.2) |
| H2 blockers | 930 | 530(57.0) | 400(43.0) | 0(0.0) |
| Proton pump inhibitors | 4,273 | 2,749(64.3) | 1,511(35.4) | 13(0.3) |
| Antianemic preparations | 1,935 | 1,151(59.5) | 781(40.4) | 3(0.2) |
| Corticosteroids | 1,125 | 627(55.7) | 497(44.2) | 1(0.1) |
| Thyroid hormones | 410 | 271(66.1) | 138(33.7) | 1(0.2) |
| Systemic antibacterials | 3,981 | 3,007(75.5) | 971(24.4) | 3(0.1) |
| Drugs for treatment of bone diseases | 484 | 178(36.8) | 303(62.6) | 3(0.6) |
| Anti-Parkinson agents | 215 | 162(75.3) | 53(24.7) | 0(0.0) |
| Antipsycotics | 1,097 | 921(84.0) | 174(15.9) | 2(0.2) |
| Anxiolytics | 2,442 | 1,794(73.5) | 639(26.2) | 9(0.4) |

ACE: angiotensin-converting enzyme

Table S12. Sensitivity Analysis : Unadjusted and Adjusted Odds Ratios for Zinc Deficiency with Comorbidities

| Zinc Deficiency  (< 60 μg/dL) | Overall (n=13,100) | | | Inpatient (n=5,614) | | | Outpatient (n=7,420) | | | Adjusted OR  by Age Group and Sex^†^ (n=13,100) | | | Adjusted OR by Age Group, Sex and Inpatient/Outpatient^‡^ (n=13,034) | | |
| --- | --- | --- | --- | --- | --- | --- | --- | --- | --- | --- | --- | --- | --- | --- | --- |
| Comorbidity | Odds Ratio^*^ | 95%CI | *p*-value | Odds Ratio^*^ | 95%CI | *p*-value | Odds Ratio^*^ | 95%CI | *p*-value | Odds Ratio^*^ | 95%CI | *p*-value | Odds Ratio^*^ | 95%CI | *p*-value |
| Intestinal infectious diseases | 1.502 | (1.280, 1.764) | <.001 | 1.097 | (0.888, 1.354) | 0.391 | 1.615 | (1.242, 2.099) | <.001 | 1.733 | (1.468, 2.045) | <.001 | 1.432 | (1.207, 1.700) | <.001 |
| Noninfective enteritis and colitis | 1.070 | (0.752, 1.522) | 0.709 | 0.967 | (0.636, 1.470) | 0.875 | 0.405 | (0.160, 1.025) | 0.057 | 1.309 | (0.910, 1.883) | 0.147 | 0.974 | (0.671, 1.413) | 0.889 |
| Tuberculosis | 1.475 | (1.163, 1.871) | 0.001 | 1.161 | (0.842, 1.601) | 0.362 | 1.566 | (1.077, 2.278) | 0.019 | 1.480 | (1.160, 1.888) | 0.002 | 1.332 | (1.035, 1.713) | 0.026 |
| Malignant neoplasms of digestive organs | 1.307 | (1.195, 1.429) | <.001 | 0.952 | (0.846, 1.072) | 0.415 | 1.212 | (1.043, 1.409) | 0.012 | 1.202 | (1.097, 1.318) | <.001 | 1.004 | (0.912, 1.105) | 0.935 |
| Nutritional anemias | 0.908 | (0.840, 0.982) | 0.015 | 1.046 | (0.927, 1.181) | 0.466 | 1.117 | (0.999, 1.248) | 0.052 | 0.929 | (0.858, 1.006) | 0.069 | 1.093 | (1.006, 1.187) | 0.036 |
| Diabetes mellitus | 0.937 | (0.867, 1.012) | 0.096 | 0.694 | (0.622, 0.774) | <.001 | 1.062 | (0.944, 1.195) | 0.315 | 0.917 | (0.847, 0.992) | 0.031 | 0.846 | (0.780, 0.919) | <.001 |
| Hypogonadism | 0.874 | (0.557, 1.371) | 0.558 | 0.800 | (0.421, 1.520) | 0.496 | 0.876 | (0.436, 1.762) | 0.710 | 0.960 | (0.606, 1.522) | 0.864 | 0.892 | (0.552, 1.443) | 0.642 |
| Short statue | 0.938 | (0.590, 1.490) | 0.785 | 0.800 | (0.421, 1.520) | 0.496 | 0.908 | (0.434, 1.902) | 0.798 | 0.996 | (0.620, 1.600) | 0.986 | 0.893 | (0.546, 1.462) | 0.654 |
| Hyperlipidemia | 1.235 | (1.096, 1.392) | <.001 | 0.742 | (0.643, 0.855) | <.001 | 1.140 | (0.883, 1.471) | 0.314 | 1.133 | (1.003, 1.281) | 0.044 | 0.799 | (0.703, 0.907) | <.001 |
| Hypertensive diseases | 1.874 | (1.714, 2.049) | <.001 | 0.922 | (0.827, 1.028) | 0.142 | 2.035 | (1.656, 2.500) | <.001 | 1.566 | (1.429, 1.717) | <.001 | 0.989 | (0.895, 1.093) | 0.826 |
| Acute myocardial infarction | 0.778 | (0.670, 0.904) | 0.001 | 0.579 | (0.473, 0.710) | <.001 | 0.919 | (0.729, 1.158) | 0.473 | 0.868 | (0.744, 1.013) | 0.073 | 0.781 | (0.667, 0.915) | 0.002 |
| Atrial fibrillation and flutter | 1.593 | (1.361, 1.864) | <.001 | 0.828 | (0.697, 0.984) | 0.032 | 2.244 | (1.430, 3.522) | <.001 | 1.215 | (1.035, 1.427) | 0.017 | 0.799 | (0.677, 0.942) | 0.008 |
| Heart failure | 1.732 | (1.582, 1.896) | <.001 | 1.144 | (1.019, 1.286) | 0.023 | 1.553 | (1.320, 1.827) | <.001 | 1.470 | (1.339, 1.613) | <.001 | 1.153 | (1.046, 1.271) | 0.004 |
| Cerebrovascular diseases | 1.643 | (1.465, 1.842) | <.001 | 1.050 | (0.915, 1.206) | 0.486 | 1.399 | (1.106, 1.770) | 0.005 | 1.376 | (1.223, 1.547) | <.001 | 1.022 | (0.904, 1.155) | 0.732 |
| Influenza and pneumonia | 1.315 | (1.194, 1.447) | <.001 | 1.028 | (0.901, 1.172) | 0.685 | 1.358 | (1.167, 1.580) | <.001 | 1.490 | (1.348, 1.648) | <.001 | 1.271 | (1.145, 1.411) | <.001 |
| COVID-19 | 1.602 | (1.481, 1.733) | <.001 | 1.097 | (0.986, 1.220) | 0.089 | 1.329 | (1.161, 1.520) | <.001 | 1.889 | (1.738, 2.054) | <.001 | 1.366 | (1.249, 1.493) | <.001 |
| Pneumonitis due to solids and liquids | 3.910 | (3.196, 4.784) | <.001 | 2.104 | (1.707, 2.593) | <.001 | 4.458 | (1.545, 12.866) | 0.006 | 2.959 | (2.410, 3.634) | <.001 | 1.858 | (1.508, 2.289) | <.001 |
| Stomatitis and related lesions | 0.637 | (0.477, 0.850) | 0.002 | 0.681 | (0.444, 1.045) | 0.079 | 0.651 | (0.426, 0.995) | 0.048 | 0.710 | (0.529, 0.952) | 0.022 | 0.717 | (0.530, 0.970) | 0.031 |
| Liver disease | 1.020 | (0.933, 1.116) | 0.657 | 1.031 | (0.903, 1.177) | 0.654 | 1.089 | (0.956, 1.241) | 0.198 | 1.242 | (1.131, 1.364) | <.001 | 1.214 | (1.102, 1.337) | <.001 |
| Dermatitis and eczema | 1.102 | (0.935, 1.298) | 0.248 | 0.890 | (0.715, 1.107) | 0.295 | 1.105 | (0.847, 1.443) | 0.462 | 1.115 | (0.943, 1.318) | 0.202 | 0.986 | (0.830, 1.173) | 0.875 |
| Alopecia areata | 0.190 | (0.087, 0.412) | <.001 | 0.330 | (0.034, 3.173) | 0.337 | 0.301 | (0.130, 0.695) | 0.005 | 0.306 | (0.139, 0.673) | 0.003 | 0.399 | (0.181, 0.879) | 0.023 |
| Decubitus ulcer and pressure area | 2.922 | (2.278, 3.748) | <.001 | 1.554 | (1.198, 2.015) | <.001 | 4.301 | (1.599, 11.565) | 0.004 | 2.403 | (1.866, 3.094) | <.001 | 1.523 | (1.180, 1.967) | 0.001 |
| Muscle wasting and atrophy, not elsewhere classified (Sarcopenia) | 2.699 | (2.389, 3.050) | <.001 | 1.436 | (1.259, 1.639) | <.001 | 1.672 | (0.857, 3.261) | 0.131 | 2.217 | (1.957, 2.512) | <.001 | 1.320 | (1.158, 1.505) | <.001 |
| Osteoporosis | 1.412 | (1.224, 1.629) | <.001 | 0.960 | (0.803, 1.148) | 0.654 | 1.391 | (1.073, 1.804) | 0.013 | 1.302 | (1.123, 1.509) | <.001 | 1.041 | (0.893, 1.213) | 0.606 |
| Chronic kidney disease | 2.133 | (1.902, 2.392) | <.001 | 1.298 | (1.126, 1.497) | <.001 | 2.471 | (2.012, 3.035) | <.001 | 1.835 | (1.632, 2.063) | <.001 | 1.471 | (1.303, 1.660) | <.001 |
| Disturbances of smell and taste | 0.369 | (0.291, 0.468) | <.001 | 0.447 | (0.242, 0.825) | 0.010 | 0.588 | (0.451, 0.765) | <.001 | 0.416 | (0.327, 0.531) | <.001 | 0.587 | (0.459, 0.750) | <.001 |
| Anorexia | 1.196 | (0.944, 1.515) | 0.138 | 0.796 | (0.600, 1.057) | 0.115 | 1.193 | (0.753, 1.889) | 0.453 | 1.042 | (0.819, 1.325) | 0.740 | 0.821 | (0.641, 1.052) | 0.119 |
| Injuries to the head | 1.753 | (1.311, 2.344) | <.001 | 0.949 | (0.685, 1.314) | 0.753 | 2.683 | (1.388, 5.190) | 0.003 | 1.541 | (1.144, 2.074) | 0.004 | 1.105 | (0.817, 1.495) | 0.517 |
| Fracture | 1.621 | (1.407, 1.867) | <.001 | 0.873 | (0.746, 1.023) | 0.093 | 1.893 | (1.314, 2.727) | <.001 | 1.330 | (1.150, 1.539) | <.001 | 0.881 | (0.758, 1.025) | 0.100 |

* For each comorbidity, individuals without the comorbidity were used as the reference group to compare with those with the comorbidity for the calculation of odds ratios.

† Adjusted Analysis: Comorbidity (with vs without), Age Group (categorized as 20s, 30s, 40s, 50s, 60s, 70s, 80 and above), and Sex are specified as covariates in logistic regression, with an interaction term included between Age Group and Sex.

‡ As the sensitivity analysis, inpatient/outpatient status was added as a covariate; however, patients with missing in-out status (n=66) were excluded from the adjusted analysis.

CI: Confidence Interval.

Table S13. Sensitivity Analysis : Unadjusted and Adjusted Odds Ratios for Zinc Deficiency with Medications

| Zinc Deficiency  (< 60 μg/dL) | Overall (n=13,100) | | | Inpatient (n=5,614) | | | Outpatient (n=7,420) | | | Adjusted OR  by Age Group and Sex^†^ (n=13,100) | | | Adjusted OR by Age Group, Sex and Inpatient/Outpatient^‡^ (n=13,034) | | |
| --- | --- | --- | --- | --- | --- | --- | --- | --- | --- | --- | --- | --- | --- | --- | --- |
| Medication | Odds Ratio^*^ | 95%CI | *p*-value | Odds Ratio^*^ | 95%CI | *p*-value | Odds Ratio^*^ | 95%CI | *p*-value | Odds Ratio^*^ | 95%CI | *p*-value | Odds Ratio^*^ | 95%CI | *p*-value |
| Antihyperglycemics | 1.077 | (0.979, 1.185) | 0.130 | 0.765 | (0.676, 0.865) | <.001 | 0.918 | (0.772, 1.093) | 0.338 | 0.955 | (0.865, 1.054) | 0.356 | 0.765 | (0.690, 0.848) | <.001 |
| Antihypertensive agents | 1.772 | (1.647, 1.907) | <.001 | 1.065 | (0.959, 1.183) | 0.242 | 1.701 | (1.514, 1.911) | <.001 | 1.467 | (1.358, 1.584) | <.001 | 1.165 | (1.074, 1.263) | <.001 |
| Spironolactone | 2.826 | (2.447, 3.264) | <.001 | 1.537 | (1.298, 1.821) | <.001 | 4.164 | (3.124, 5.549) | <.001 | 2.523 | (2.179, 2.922) | <.001 | 1.909 | (1.641, 2.221) | <.001 |
| Furosemide | 2.552 | (2.321, 2.807) | <.001 | 1.430 | (1.271, 1.608) | <.001 | 2.988 | (2.494, 3.581) | <.001 | 2.138 | (1.939, 2.357) | <.001 | 1.606 | (1.450, 1.779) | <.001 |
| ACE inhibitors | 1.215 | (0.969, 1.523) | 0.092 | 0.906 | (0.689, 1.191) | 0.479 | 0.968 | (0.618, 1.516) | 0.888 | 0.974 | (0.775, 1.225) | 0.822 | 0.810 | (0.640, 1.026) | 0.081 |
| Angiotensin II receptor blockers | 1.131 | (1.022, 1.252) | 0.017 | 0.813 | (0.708, 0.933) | 0.003 | 1.281 | (1.091, 1.504) | 0.002 | 0.987 | (0.889, 1.095) | 0.806 | 0.908 | (0.815, 1.011) | 0.078 |
| Antihyperlipidemics | 0.750 | (0.679, 0.830) | <.001 | 0.669 | (0.582, 0.768) | <.001 | 0.736 | (0.628, 0.863) | <.001 | 0.664 | (0.599, 0.736) | <.001 | 0.645 | (0.580, 0.717) | <.001 |
| Statins | 0.775 | (0.697, 0.862) | <.001 | 0.701 | (0.606, 0.811) | <.001 | 0.731 | (0.616, 0.868) | <.001 | 0.685 | (0.614, 0.764) | <.001 | 0.659 | (0.589, 0.738) | <.001 |
| Antithrombotic agents | 1.953 | (1.808, 2.110) | <.001 | 0.918 | (0.827, 1.019) | 0.108 | 2.125 | (1.844, 2.448) | <.001 | 1.621 | (1.496, 1.757) | <.001 | 1.093 | (1.001, 1.193) | 0.047 |
| H2 blockers | 1.154 | (1.005, 1.324) | 0.042 | 0.809 | (0.676, 0.968) | 0.021 | 1.299 | (1.036, 1.628) | 0.023 | 1.108 | (0.962, 1.275) | 0.154 | 0.956 | (0.827, 1.105) | 0.541 |
| Proton pump inhibitors | 1.752 | (1.625, 1.890) | <.001 | 1.035 | (0.932, 1.149) | 0.516 | 1.634 | (1.440, 1.853) | <.001 | 1.553 | (1.436, 1.678) | <.001 | 1.176 | (1.082, 1.277) | <.001 |
| Antianemic preparations | 2.226 | (2.019, 2.454) | <.001 | 1.375 | (1.206, 1.566) | <.001 | 2.903 | (2.490, 3.385) | <.001 | 2.027 | (1.835, 2.239) | <.001 | 1.780 | (1.607, 1.973) | <.001 |
| Corticosteroids | 1.453 | (1.284, 1.645) | <.001 | 0.936 | (0.793, 1.105) | 0.437 | 1.903 | (1.570, 2.307) | <.001 | 1.504 | (1.325, 1.708) | <.001 | 1.307 | (1.146, 1.491) | <.001 |
| Thyroid hormones | 2.056 | (1.688, 2.504) | <.001 | 1.599 | (1.245, 2.054) | <.001 | 1.685 | (1.178, 2.411) | 0.004 | 1.864 | (1.524, 2.280) | <.001 | 1.548 | (1.257, 1.906) | <.001 |
| Systemic antibacterials | 2.615 | (2.421, 2.825) | <.001 | 1.484 | (1.336, 1.650) | <.001 | 2.172 | (1.882, 2.508) | <.001 | 2.419 | (2.235, 2.617) | <.001 | 1.676 | (1.535, 1.828) | <.001 |
| Drugs for treatment of bone diseases | 0.960 | (0.792, 1.162) | 0.673 | 0.944 | (0.700, 1.273) | 0.706 | 1.121 | (0.859, 1.462) | 0.400 | 0.849 | (0.698, 1.033) | 0.102 | 0.948 | (0.774, 1.161) | 0.605 |
| Anti-Parkinson agents | 1.676 | (1.279, 2.196) | <.001 | 0.916 | (0.670, 1.253) | 0.584 | 2.579 | (1.494, 4.451) | <.001 | 1.494 | (1.134, 1.968) | 0.004 | 1.121 | (0.846, 1.485) | 0.426 |
| Antipsychotics | 1.556 | (1.373, 1.763) | <.001 | 0.838 | (0.728, 0.966) | 0.015 | 1.602 | (1.160, 2.211) | 0.004 | 1.411 | (1.242, 1.603) | <.001 | 0.904 | (0.792, 1.033) | 0.140 |
| Anxiolytics | 1.554 | (1.421, 1.700) | <.001 | 0.830 | (0.742, 0.929) | 0.001 | 1.666 | (1.398, 1.985) | <.001 | 1.372 | (1.251, 1.503) | <.001 | 0.958 | (0.869, 1.057) | 0.392 |

* For each medication, individuals without the medication were used as the reference group to compare with those with the medication for the calculation of odds ratios.

† Adjusted Analysis: Medication (with vs without), Age Group (categorized as 20s, 30s, 40s, 50s, 60s, 70s, 80 and above), and Sex are specified as covariates in logistic regression, with an interaction term included between Age Group and Sex.

‡ As the sensitivity analysis, inpatient/outpatient status was added as a covariate; however, patients with missing in-out status (n=66) were excluded from the adjusted analysis.

ACE: angiotensin-converting enzyme, CI: Confidence Interval.
